# Selective Analysis of Redox Processes at the Electrode Interface with Time-Resolved Raman Spectroscopy

**DOI:** 10.1021/acs.langmuir.3c00633

**Published:** 2023-07-21

**Authors:** W. J. Niels Klement, Jorn D. Steen, Wesley R. Browne

**Affiliations:** † Molecular Inorganic Chemistry, Stratingh Institute for Chemistry, Faculty of Science and Engineering, University of Groningen, Nijenborgh 4, 9747 Groningen, AG, The Netherlands; ‡ Pharmaceutical Analysis, Groningen Research Institute of Pharmacy, University of Groningen, Antonius Deusinglaan 1, 9700 Groningen, AD, The Netherlands

## Abstract

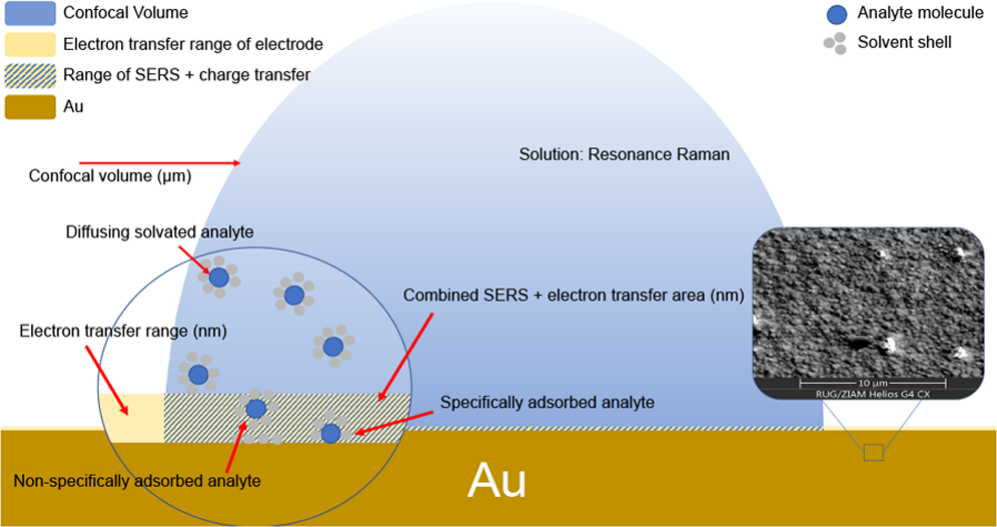

Electrochemistry and electrochemical reactions are increasingly important in the transition to a sustainable chemical industry. The electron transfer that drives such reactions takes place within nanometers of the electrode surface, and follow-up chemical reactions take place within the diffusion layer. Hence, understanding electrochemical reactions requires time-, potential-, and spatially resolved analysis. The confocal nature of Raman spectroscopy provides high spatial resolution, in addition to detailed information on molecular structure. The intrinsic weakness of nonresonant Raman scattering, however, is not sensitive enough for relatively minor changes to the solution resulting from reactions at the electrode interface. Indeed, the limit of detection is typically well above the concentrations used in electrochemical studies. Here, we show that surface-enhanced Raman scattering (SERS) and resonance Raman (rR) spectroscopy allow for spatially and time-resolved analysis of solution composition at (<1–2 nm) and near (within 5 μm) the electrode surface, respectively, in a selective manner for species present at low (<1 mM) concentrations. We show changes in concentration of species at the electrode surface, without the need for labels, specific adsorption, or resonance enhancement, using a SERS-active gold electrode prepared readily by electrochemical surface roughening. A combination of smooth and roughened gold electrodes is used to distinguish between surface and resonance enhancement using the well-known redox couples ferrocene and 2,2’-azino-bis(3-ethylbenzothiazoline-6-sulfonic acid) (ABTS). We discuss the impact of specific adsorption on the spectral analysis with the ruthenium(II) polypyridyl complex, [Ru(bpy)_3_]^2+^. The dual function of the electrode (surface enhancement and electron transfer) in the analysis of solution processes is demonstrated with the reversible oxidation of TMA (4,*N,N*-trimethylaniline), where transient soluble species are identified in real time, with rapid spectral acquisition, making use of localized enhancement. We anticipate that this approach will find use in elucidating electro(catalytic) reactions at electrode interfaces.

## Introduction

The current demands on electrochemical materials, sustainable energy, electrocatalysis, and synthetic applications of electrochemistry place increasing attention on the reactions that occur at electrode interfaces.^1−9^ Heterogeneous electron transfer requires redox-active molecules to approach the Stern layer. Subsequent chemical reactions, for example, comproportionations and molecular rearrangements, take place further from the electrode, typically within the Nernst diffusion layer.^10^ The concentration of species, as well as the conditions (e.g., pH),^11^ at and near the surface of the electrode can differ substantially from that of the bulk solvent. Indeed, there is a dependence on time, distance, and potential that is not typically encountered in homogeneous reactions.^12^ Hence, spatially resolved analysis is required to study electrochemical reactions at (<1–2 nm) the electrode interface.

The confocality achievable with Raman spectroscopy, due to the use of visible and near-infrared (NIR) light, lends itself particularly well to achieve relatively high spatial resolution (e.g., <5 μm).^13^ However, this confocality is still insufficient to selectively probe the region close (<1–2 nm) to the electrode surface. Furthermore, the intensity of Raman scattering is linearly dependent on concentration. The limits of detection are typically in the 50 mM range depending on the analyte, which is well above the 0.1–2 mM concentrations used in analytical electrochemistry. An advantage, however, is that the spectra obtained are highly sensitive to molecular structure, which enables analysis of complex mixtures of compounds. Furthermore, selective enhancement of Raman scattering from species of interest can overcome the concentration-related limitations, namely, resonance Raman (rR) and surface-enhanced Raman scattering (SERS).

Resonance enhancement requires that the wavelength of the laser used coincides with an electronic absorption band (i.e., is resonant) of the analyte of interest.^14^ This requirement is not routinely satisfied with NIR Raman systems (typically, at 632.8 or 785 nm). However, one-electron oxidized and reduced species are often open shell and show strong low-energy absorption bands that are resonant with NIR lasers. Hence, resonance Raman spectroscopy is useful, for example, in the study of redox polymers.^3,15,16^

An alternative and more generally applicable approach is surface enhancement (SERS) through interaction of molecules with the surface plasmon resonance of nanoparticles and metal surfaces. Importantly, the molecule–surface proximity required (<1–2 nm) for optimal^17^ surface enhancement of Raman scattering^12,18−21^ is similar to the distance required for heterogeneous electron transfer.^10^

The proximity to the surface required in SERS means that most studies to date have focused on self-assembled monolayers (SAMs)^5,18,21,22^ and polymers^15,16,23^ on electrode surfaces. Recording SERS spectra concomitant with changing electrode potential is referred to as electrochemical SERS (EC-SERS) or SERS spectroelectrochemistry. This approach has been widely applied to the study of electrochemical processes,^24−26^ from identification of compounds^7^ to the analysis of biological matrices.^5,27,28^ EC-SERS has been applied, for example, to follow changes in redox state and adsorption of anions in ferrocene-based SAMs, as a means to understand the effect of ions on their cyclic voltammetry,^29^ as well as to study electrochemical processes in SAMs.^22^ Indeed, the combination of SAMs and SERS can be particularly useful to study reactions, such as the oxidatively driven aryl–aryl coupling of nitrospiropyran SAMs on roughened gold substrates.^30^

Silver, platinum, and gold are commonly used electrode materials that can be roughened to enable SERS spectroscopy^21,31,32^ and at the same time act as a working electrode. Electrochemical roughening of these surfaces through oxidation/reduction cycles is generally sufficient to provide SERS activity.^33^ Mostly, electrochemically roughened gold and silver are used to study the electrochemical behavior of self-assembled monolayers (SAMs).^34^ The wider potential range and facile electrochemical roughening of gold and, to a lesser extent, platinum make these metals generally more versatile as electrode materials for EC-SERS than silver. Electrodes used for acquisition of SERS spectra during voltammetry (EC-SERS) include gold-on-glass substrates,^25^ gold-coated scanning tunneling microscopy (STM) and atomic force microscopy (AFM) tips (TERS),^35,36^ and screen-printed SERS-active electrodes in which (silver) nanoparticles are incorporated to generate surface enhancement on an electrode support.^26^ The approach described by Sanger et al. uses a screen-printed nanostructured working electrode that is also SERS active.^25^ Although effective, a readily accessible approach to a reliable SERS-active electrode is desirable, such as that obtained by electrochemical roughening of gold electrodes. Furthermore SAMs formed spontaneously by analytes with gold-binding groups (chemisorption) increase local concentrations and are thus not suitable for analysis where the behavior of species in solution is of interest. Additionally, while detection of a single analyte at an interface is most widely of concern, the study of dynamic redox processes over time, while they are happening, at the electrode surface offers potential insight into where changes to the composition of analytes occur.

Although SERS using aggregated silver and gold colloids is used widely to obtain spectra in solution, the detection of solutes at roughened electrode surfaces has been reported sparsely due to the low effective surface concentration realized with the millimolar concentration solutions of analytes typically used for cyclic voltammetry (1 mM bulk concentration corresponds to an effective surface concentration of 10^–13^ mol cm^–2^, with the surface defined as the volume within 1 nm of the electrode surface). Furthermore, the physical adsorption of analytes and other species (e.g., impurities) on SERS-active surfaces can easily result in domination of the SERS-enhanced spectrum over that of the soluble analyte of interest.

**Figure 1:**
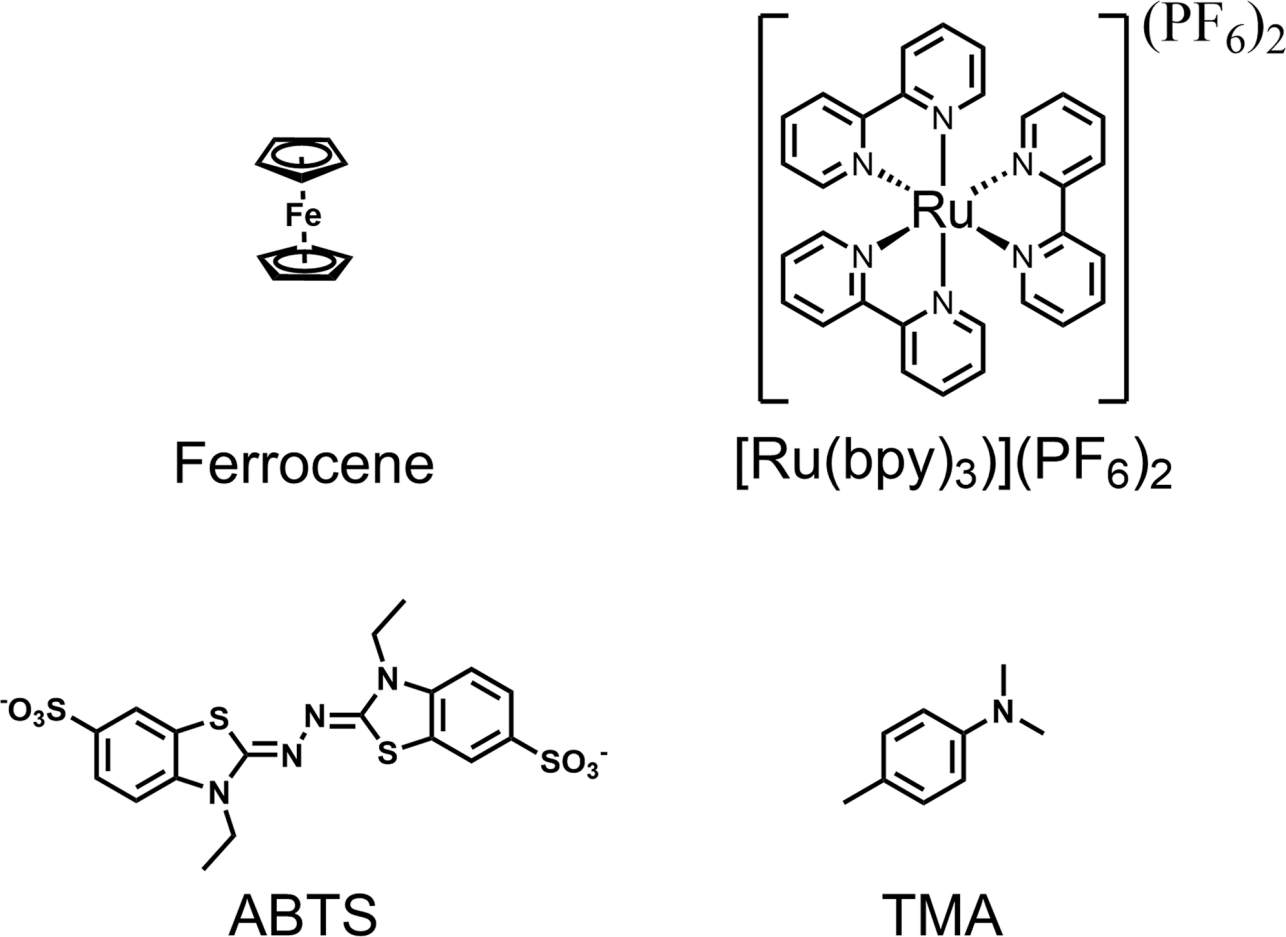
Structures of compounds discussed in the text.

It is well noted in the literature that while a <1–2 nm distance from the SERS surface is optimal^17^ for SERS enhancement and intensities, SERS signals originating from molecules up to 30 nm distant from the surface can be observed, although at a much lower intensity.^21^ In the present contribution, we show that smooth and electrochemically roughened gold surfaces can be employed effectively both as a working electrode and, in the latter case, as a SERS substrate to study (electro)chemical reactions of soluble species by Raman microspectroscopy. We show the general applicability of the method through the analysis of the example compounds ferrocene; 2,2’-azino-bis(3-ethylbenzothiazoline-6-sulfonic acid) (**ABTS**); and 4,*N*,*N*-trimethylaniline (**TMA**), Figure 1.

Ferrocene, a common probe in redox chemistry,^37,38^ undergoes reversible oxidation to the ferrocenium ion within the accessible potential range of gold^22,39^ and has been used in earlier EC-SERS studies of SAMs.^22^ Importantly, although ferrocene and ferrocenium can show resonance enhancement with excitation <700 nm, enhancement is not observed in solution with excitation at 785 nm. The impact of interference due to adsorption is probed with the well-known complex [Ru(bpy)_3_]^2+^. **ABTS** (2,2’-azino-bis(3-ethylbenzothiazoline)-6-sulfonic acid), a commonly used probe for oxidations in biochemical contexts, shows resonance enhancement in its oxidized state at commonly applied laser wavelengths (632.8 and 785 nm),^40^ which allows for the combination of SERS and resonance enhancement to be explored. Finally, analysis of a redox-active organic compound 4,*N*,*N*-trimethylaniline (**TMA**) is described. **TMA** is selected to demonstrate how SERS spectroscopy can be used to follow electrochemically induced changes and processes at the electrode. TMA lacks absorption in the near-infrared (NIR), and consequently does not show resonance enhancement, in any of its redox states. We detect local changes in pH during voltammetry by changes in the SERS spectrum, similar to earlier studies on thiophenol SAMs.^11,23,41−43^

## Experimental Section

Solvents and reagents were purchased from Sigma-Aldrich and used as received. Ferrocenium hexafluorophosphate was prepared by oxidation of ferrocene with Fe(III)Cl_3_ in acetone/water, followed by precipitation with KPF_6_ and recrystallization from acetone/water. [Mn_2_^III^O_3_(TMTACN)_2_](PF_6_)_2_ and [Ru(bipy)_3_](PF_6_)_2_ were prepared by standard methods.^44,45^

Raman spectra at 785 nm were obtained with a home-built Raman microscope (Figure S2) using a ONDAX LM-785 laser (75 mW). A picture of the setup is available in the SI (Figure S2). The power of which was reduced to ca. 1 mW at the sample, unless indicated otherwise, using a half-waveplate (Thorlabs, WPMQ10M-780) to rotate laser polarization combined with a polarizing beam splitter (Thorlabs, CCM1-PBS252/M). A laser power at a sample of 1 mW was used for all Raman and SERS measurements unless indicated otherwise. The beam splitter was followed by beam expansion with a pair of planoconvex lenses (5 and 7.5 cm) and a second half-waveplate to control polarization direction before aligning with the optical access of the spectrometer using a 45° dichroic (Semrock, LPD02-785RU-25) directed to the objective lens of the BX51 microscope via gold-coated beam steering mirrors (Figure S1). The spectra were collected in 180° backscattering mode with a long-pass Rayleigh line rejection filter (Semrock, LP02-785RU-25) and focused with a 3.5 cm focal length planoconvex lens into a Kymera-193 spectrograph with an iDus-420-BU CCD detector and 600 lines/mm 830 nm blaze grating. Spectral calibration was carried out using the spectrum of polystyrene or cyclohexane (ASTM E1840-96(2014)). Spectra were processed using SpectraGryph-on v.1.2.15 for, e.g., baseline correction and normalization and were plotted using Python.

Gold beads were fabricated from a gold wire (0.5 mm diameter, Thermo Scientific), using a butane/air torch to melt the wire at one end to form a bead. The relatively smooth surface was used either without roughening (non-SERS active) or roughened following the procedure of Liu et al.^33^ (the roughening method used is described in the SI, Figure S3). Scanning electron microscopy images were obtained with a Thermo Fisher Helios G4 CX SEM, with samples mounted on an aluminum stage with immobilization via the electrode stem with silver epoxy glue. Electrochemical analyses were performed using a CH Instruments potentiostat (CHI1200, CHI604e), with a gold working, platinum wire counter, and Ag/AgCl reference electrode. A picture of the electrochemical cell is available in the SI, Figure S1. 0.1 M potassium hexafluorophosphate (KPF_6_) or tetrabutylammonium hexafluorophosphate (TBAPF_6_) was used as the electrolyte in aqueous and organic solvents, respectively, unless indicated otherwise. Note that glassware and all material in contact with solutions and electrodes were cleaned aggressively with KMnO_4_ in conc. H_2_SO_4_ followed by pure water and a 1:1:1 mixture of conc. HCl/H_2_O_2_/H_2_O. **Caution: these mixtures must not be used in proximity with organic solvents or oxidizable materials**. The ruthenium complex [Ru(bpy)_3_]^2+^, where bpy = 2-2’-bipyridine, was used to assess the relative SERS performance and activity of roughened electrodes.

## Results and Discussion

**Figure 2:**
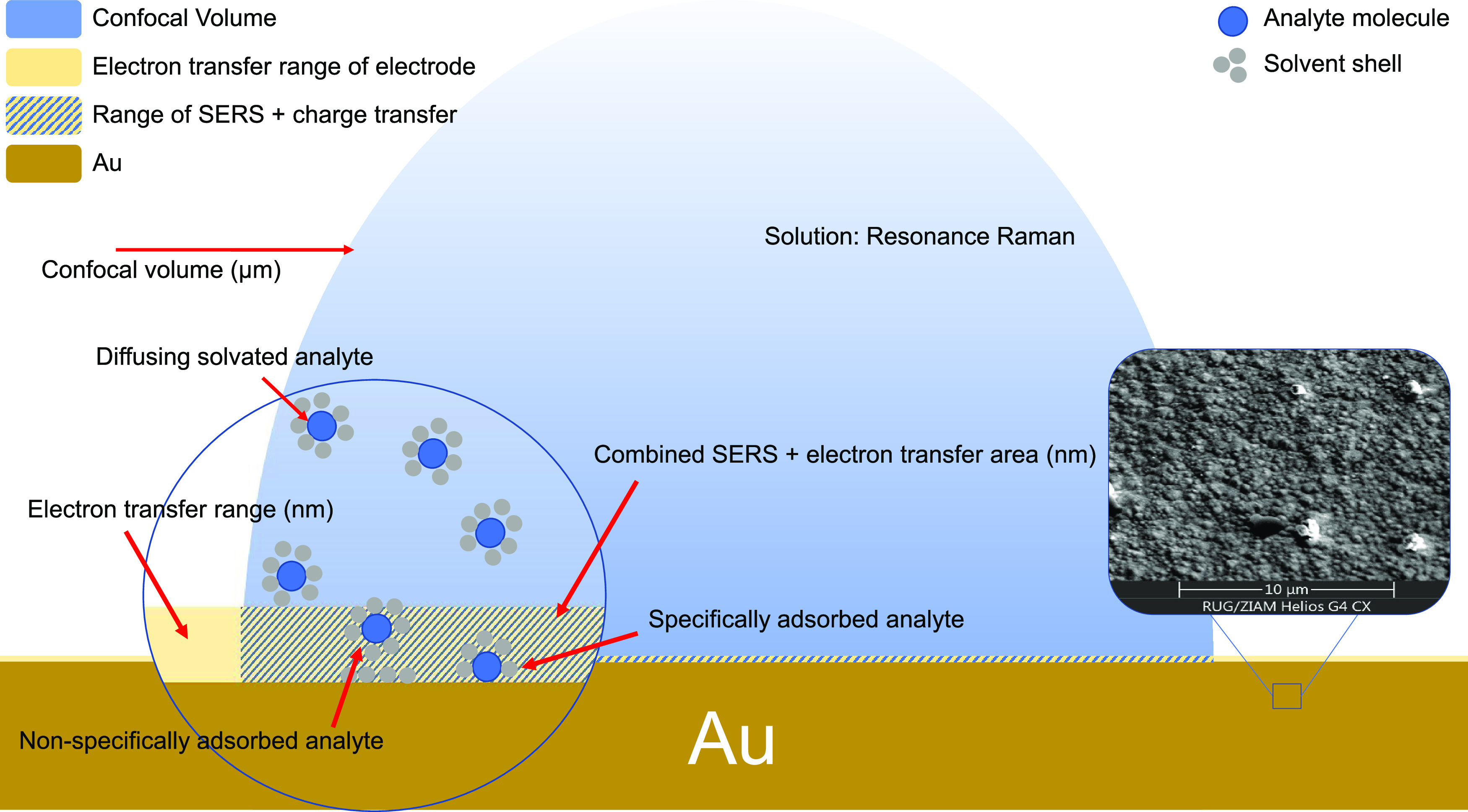
Depiction of the confocal volume of the Raman microscope at the gold surface. Inset: SEM image of the roughened gold surface. Further SEM images are available in the SI (Figure S4).

The experimental arrangement for electrochemical Raman microspectroscopy was optimized for surface analysis. Specifically, the z-confocality of the microscope was increased by expansion of the laser beam to improve the relative contribution of the ‘surface’ to the Raman spectra obtained.^2^ Nevertheless, molecules at the surface (within a few nm) contribute only a fraction (<0.1%) of the total Raman scattering collected from the confocal volume compared to the solvent and any analytes in the remaining few microns from the electrode (Figure 2). Hence, in the absence of enhancement mechanisms, such as SERS and resonance enhancement, the spectrum obtained even with the gold surface in the focal plane of the microscope is essentially that of the electrolyte. Importantly, when the confocal volume contains the surface of the electrode, by default the region in which charge transfer occurs coincides with the SERS-active region.

### SERS Spectroelectrochemistry of Ferrocene

**Figure 3:**
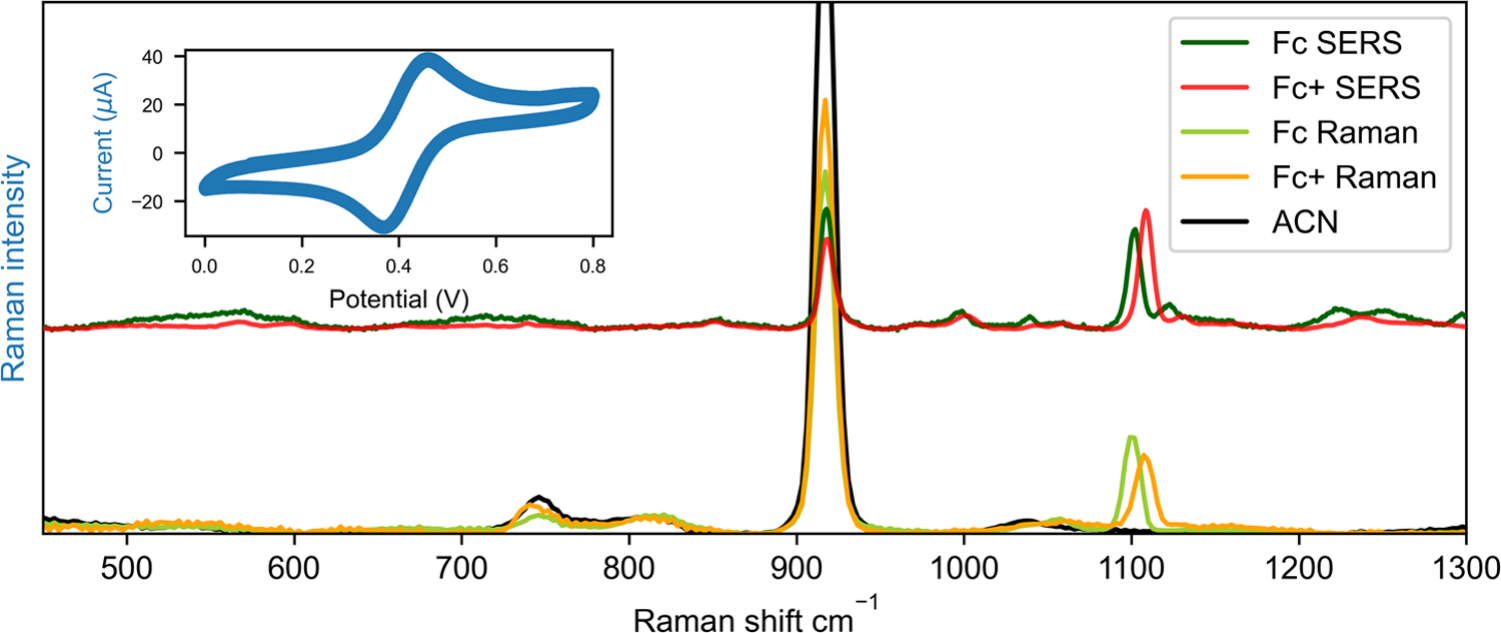
Comparison between SERS and Raman spectra. Surface-enhanced Raman spectra (λ_exc_ 785 nm) of ferrocene (1 mM) (Fe(II), green) and ferrocenium (Fe(III), red) in acetonitrile, measured at a roughened gold electrode at 0.1 and 0.8 V, compared to Raman spectra (λ_exc_ 785 nm) of ferrocene (100 mM) (light green) and ferrocenium (100 mM) (orange) in acetonitrile. Inset shows corresponding cyclic voltammogram of ferrocene, scan rate of 0.1 V/s, with a Pt counter and Ag/AgCl reference electrode. Exposure time was 0.5 s. The potential was kept constant during spectral acquisition.

The redox chemistry and Raman spectroscopy of ferrocene are well-studied^29,46−49^ and reviewed.^50^ Electrochemical SERS (EC-SERS) of a self-assembled ferrocene-containing monolayer on a roughened gold electrode has been reported.^22^ Ferrocene can undergo a reversible single electron oxidation (Fe^II^/Fe^III^) at low positive potentials (0.4 V vs SCE). Ferrocene and ferrocenium are colored and show weak resonance enhancement of Raman scattering upon excitation at visible wavelengths.^51^ At 785 nm, the Raman spectra of both are fully nonresonant, with a similar Raman scattering cross section for most bands in both oxidation states (Figures S7 and S8). The limit of detection under the optical conditions used for SERS studies (vide infra) was found to be 20 mM (with 2 s exposure time and 3.3 mW laser power (Figure S9)). A lower limit of detection can be achieved with longer acquisition times/higher laser power. However, with the conditions and concentrations used for the SERS study below, 10 mM, contributions from nonresonant Raman scattering can be excluded. Therefore, at the concentrations used in cyclic voltammetry (1 mM), nonresonant Raman scattering from the complex is too weak to be detected, and only signals enhanced by interaction with the surface contribute to the Raman spectra obtained (Figure 3). The SERS spectra of ferrocene at a roughened gold bead electrode show initially predominantly bands attributable to solvent (at 379, 919, and 1374 cm^–1^) and, on occasion, additional bands due to impurities adsorbed on the surface (Figure S10). The Raman spectrum recorded with the roughened gold bead in the confocal volume of the Raman microspectrometer over time with an incremental increase in the concentration of ferrocene shows a brief period of increase followed by a constant Raman intensity, Figure S11. Furthermore, the rate of decrease in Raman intensity following a decrease in concentration by dilution was comparable to the initial rate of increase (Figure S12). The data indicate that adsorption is not significant under these conditions.

**Figure 4:**
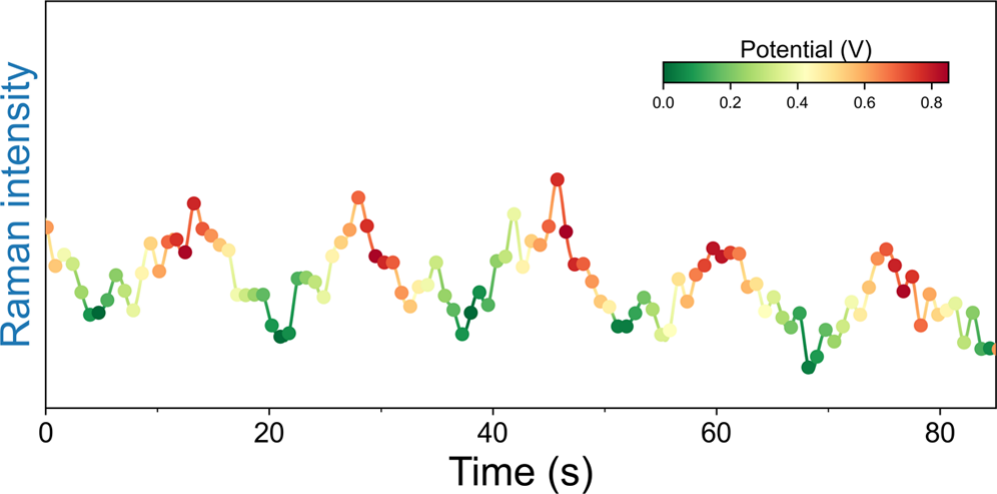
Integrated intensity of the band at 1113 cm^–1^ of ferrocenium, recorded during voltammetric cycles. Green to red color gradients indicate the potential. The periodic increase and decrease of Raman intensity is in phase with voltammetric cycles. Exposure time per spectrum was 100 ms, corresponding to 1 spectrum for each 10 mV range in the voltammogram.

At positive overpotentials, the characteristic band of ferrocenium at 1113 cm^–1^ is observed,^29^ the intensity of which increases with the applied potential. The changes in the spectrum over time during cyclic voltammetry are periodical, reversible, and track the change in potential well, over at least 100 cycles, most clearly for the band at 1113 cm^–1^ (Figure 4). Bands due to impurities adsorbed to the roughened bead did not appear periodically and could be excluded from analysis therefore. The contribution of these randomly appearing Raman bands increases with lower acquisition times, where they can dominate an individual spectrum. The frequency of such events decreases over time and voltage cycles, as expected, due to oxidative surface cleaning (Figure S13).^52,53^ Periodically recurring bands in the SERS spectra were identified by comparison with nonresonant reference spectra (Figures S7 and S8).

The Raman band chosen for analysis, 1113 cm^–1^ of the ferrocenium ion (**Fc**^+^), was used earlier in EC-SERS^22^ and is assigned to the asymmetric ring breathing of the cyclopentadienyl ring.^47^ The behavior observed here is consistent with changes in concentration of ferrocene and ferrocenium at the electrode–solution interface during cyclic voltammetry.^29^ Notably, the relatively constant intensity of Raman bands of acetonitrile at 919 cm^–1^, compared to reference (non-SERS) measurements, shows that the Raman bands of the electrolyte and solvent are not significantly enhanced, Figure S14. The data reported here confirm that surface enhancement of Raman scattering from solutes can be sufficient to track the oxidation state of species at the electrode provided care is taken to exclude contributions from specifically adsorbed species.

### Interference from Specific Adsorption

**Figure 5:**
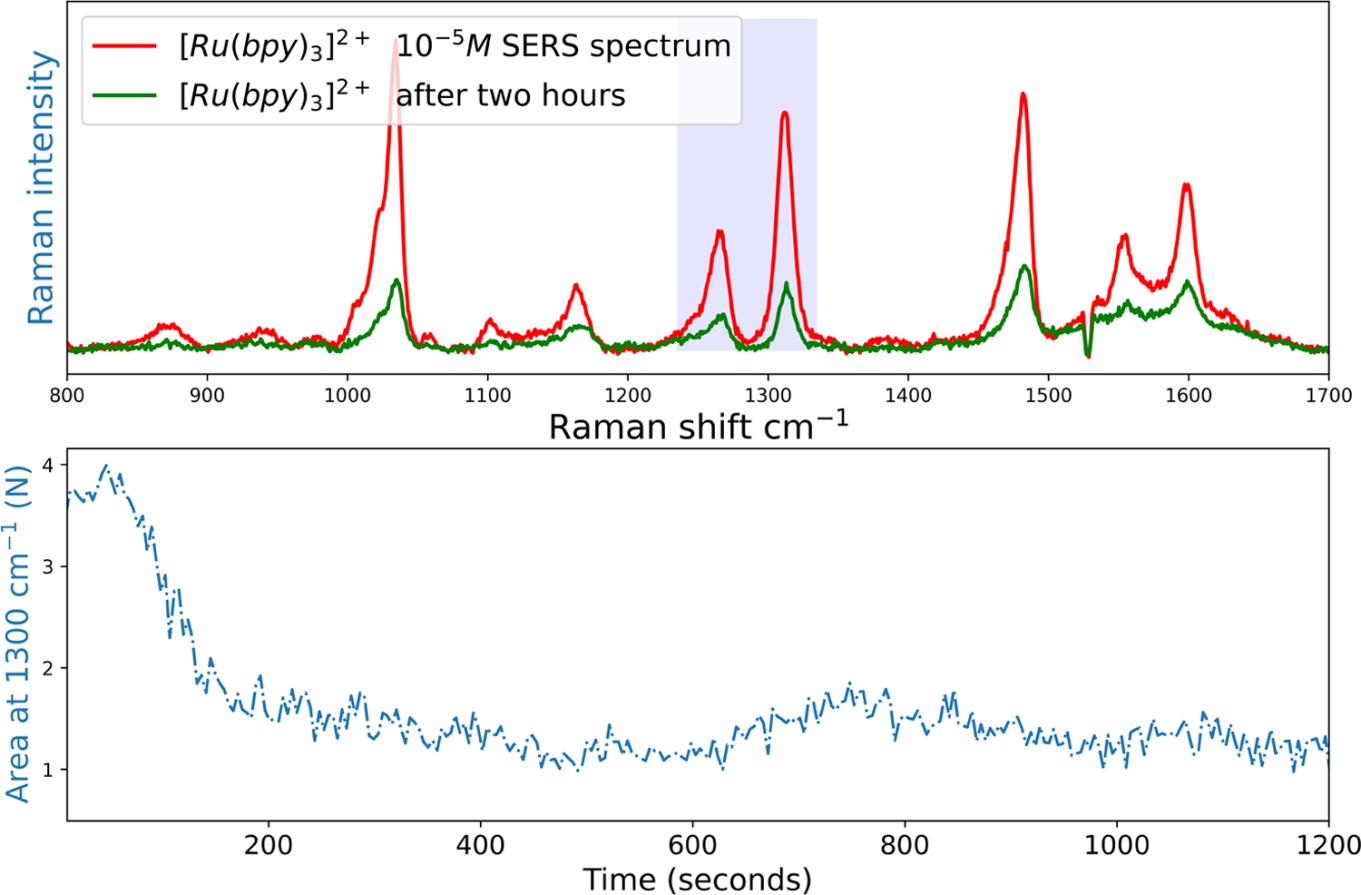
(Top) SERS spectrum (λ_exc_ 785 nm) of [Ru(bpy)_3_]^2+^ (10 μM) in water and of the same bead after standing subsequently for two hours in neat water. Exposure time is 0.5 s. (Bottom) Integrated intensity of band at 1300 cm^–1^ (blue highlight on top), the decrease in signal over time corresponds to partial desorption of [Ru(bpy)_3_]^2+^ from the SERS substrate.

Specific adsorption of species on electrodes is a common phenomenon when working with noble metals, in particular for sulfur-containing molecules,^7,27,35,54^ but also with aromatic compounds.^2,23,25,33,36^ Specific and nonspecific adsorption can be defined (following recommendation by IUPAC) as follows: Specific adsorption of a species implies direct contact with the gold atoms and partial loss of its solvation sphere. Nonspecific adsorption refers to contact of a fully solvated ion with the solvent layer (Helmholtz plane) at the electrode. This specifically adsorbed species can impact the SERS spectra obtained as shown in the previous section. The extent of specific adsorption of species on electrode surfaces and the resulting contribution to Raman intensity on the electrode surface is difficult to predict. Furthermore, it is difficult to distinguish between a SERS signal originating from a molecule in solution and from one adsorbed to the electrode surface. It has been proposed that adsorption can result in changes in the measured Raman spectrum due to, for example, nonisotropic orientation of the adsorbed molecules.^55^ Adsorption can heavily influence the SERS spectra obtained in that the adsorption increases the effective concentration of the species under study at the electrode. Therefore, adsorption needs to be accounted for. The complex [Ru(bpy)_3_]^2+^ is a case in point. The complex is cationic and does not contain functional groups that are known to bind to gold. Its SERS spectrum is equivalent to its nonresonant Raman spectrum and so can be used to demonstrate the impact of specific adsorption on Raman spectra (Figure 5).^56,57^ A roughened (SERS-active) electrode was first immersed in a solution of [Ru(bpy)_3_]^2+^ (10 μM in water) and was subsequently removed and immersed in pure water while the Raman scattering intensity from the electrode was monitored over time. The Raman bands of [Ru(bpy)_3_]^2+^ initially decrease in intensity, consistent with desorption of nonspecificially adsorbed species, but the residual signal remains stable for several hours, which is consistent with (specific) adsorption to the electrode. Determination of surface coverage is challenging for weakly adsorbed species. Electrochemical determination is hampered by the high redox potential of [Ru(bpy)_3_]^2+^ (1.25 V vs SCE), which coincides with that of Au/[Au(CH_3_CN)_2_]_3_^+^. [Os(bpy)_3_]^2+^ (Figure S16) is chemically and vibrationally equivalent to [Ru(bpy)_3_]^2+^ but its redox potential is ca. 0.8 V and hence cyclic voltammetry can be used to estimate surface coverage. The surface coverage with [Os(bpy)_3_]^2+^ and SERS spectra before and after voltammetry were obtained using a smooth and a roughened gold bead electrode (Figure S17). The SERS spectrum before and after voltammetry was similar (albeit weaker after voltammetry, consistent with the 10-fold decrease of compound from the surface) and obtained readily in air with the roughened gold bead. Raman bands were not observed with the smooth gold bead, indicating that the observed spectrum obtained with the roughened bead is due to surface enhancement. For both, integration of the first and last oxidation waves in the multicyclic voltammogram indicates a surface density of 10^–10^ and 10^–11^ mol cm^–2^, respectively (Figures S18 and S19). Hence, we can conclude that the SERS spectra are obtained readily for physisorbed species even at submonolayer surface coverage.

In the present study, the relative intensity of bands of analytes examined (ferrocene/ ferrocenium, **ABTS**, and **TMA**) is the same for both the surface and the resonance-enhanced spectra as for their nonresonant Raman spectra (vide infra). The lack of spectral differences indicates chemosorption is not significant for these compounds. Furthermore, specific adsorption is clearly occurring with [Ru(bpy)_3_]^2+^ and [Os(bpy)_3_]^2+^, manifested, e.g., in the persistence of signal in both SERS and cyclic voltammetry. Such persistence is not observed for the other compounds. Indeed, e.g., **TMA** readily washes off the SERS substrate when the solvent is exchanged, Figure S15. The lack of persistent surface interaction is consistent with the absence of specific adsorption by these species, and hence the interaction is nonspecific at most. At least, the species are transiently present at or near the surface on the time scale of our measurements and are interacting dynamically enough with the environment of the bulk solution, such as also seen for ferrocene in Figure S9. Overall, it is concluded that specific adsorption is not evident in the present case for ferrocene, **ABTS**, and **TMA**. As a final remark, the specific adsorption of [Ru(bpy)_3_]^2+^ and the resulting SERS spectrum are useful in confirming the SERS activity of electrodes after adsorption tests for the other analytes.

### Raman Spectroelectrochemistry of ABTS at a Gold Electrode

**Scheme 1:**
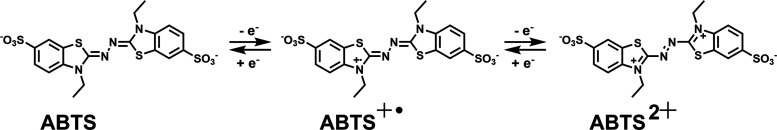
Redox States of ABTS Note that although ABTS is a dianion in solution due to the −SO_3_^–^ substituents, the radical and doubly oxidized states are indicated as cation and dication for clarity.

**ABTS** is used widely to probe electron-transfer oxidations in both biology and chemistry.^40,58,59^ Its ease of handling, as well as the characteristic and easily observed NIR absorption band (ϵ > 14 × 10^3^ M^–1^ cm^–1^, Figure S20)^58^ of the radical monocation (**ABTS**^+•^), formed by electron-transfer oxidation (Scheme 1), means it is used extensively in assays, such as for determining the activity of peroxidases.^59^ The radical monocation (**ABTS**^+•^), electrochemically generated by preparative oxidation at a carbon electrode, shows the expected resonant enhancement of its Raman scattering at 785 nm,^40^ with its characteristic triplet of bands between 1350 and 1500 cm^–1^ (Figure S21). The absorption band of **ABTS**^+•^ at 785 nm, together with the polarizability of the extended π-system, results in strong resonance enhancement of its Raman scattering. The combination of resonance enhancement^40^ and surface enhancement^60^(i.e., SERS) that is encountered at λ_exc_ 785 nm is used here. A differentiation is made between resonance Raman (rR) and surface-enhanced resonance Raman scattering (SERRS). The former comes from **ABTS**^+•^ present in the whole confocal volume of the Raman spectrometer, and the latter includes contributions from molecules that interact with the surface of the roughened gold electrode.

**Figure 6:**
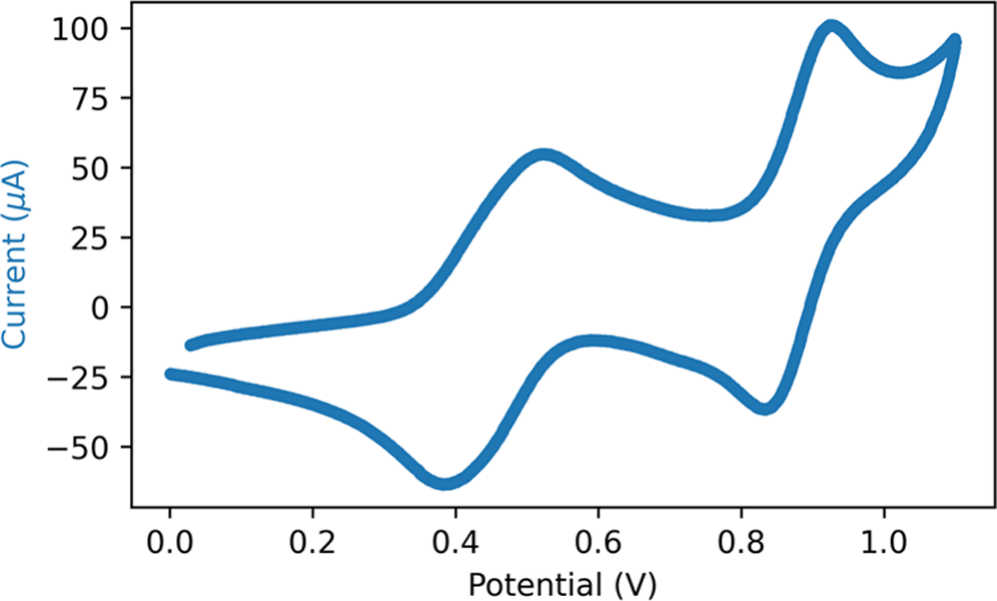
Cyclic voltammogram of **ABTS** (0.65 mM) in water (0.1 M KPF_6_). A gold working, Pt counter, and Ag/AgCl reference electrode. Scan rate is 0.1 Vs^–1^.

**Figure 7:**
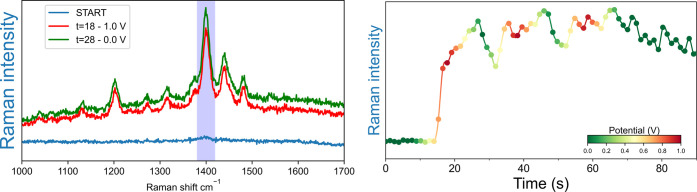
(Left) Resonance Raman spectra (λ_exc_ 785 nm) of **ABTS**^+•^, recorded at 0 V (blue), 1.0 V (red), and 0.0 V again (green) during cyclic voltammetry at an unroughened gold bead as a working electrode. Exposure time is 1 s. (Right) Integrated area of the band at 1400 cm^–1^ during several cycles shows periodic increase and decrease in intensities with each potential cycle.

The electrochemical oxidation of **ABTS** with a nonroughened gold electrode shows the expected pair of redox waves for the first and second oxidation (Figure 6). The Raman spectrum obtained while focused on the electrode surface (i.e., the confocal volume of the Raman microscope contains the surface) shows only Raman scattering from water, as expected considering the low concentration of **ABTS** present (Figure 7). Interestingly, the characteristic resonantly enhanced bands of **ABTS**^+•^ at 1400 cm^–1^ do not appear immediately when the potential applied exceeds 0.5 V. Only as the electrode is polarized to 1.0 V vs Ag/AgCl, on the initial sweep of a cyclic voltammogram, does the intensity increase, initially rapidly, after which it varies only moderately over further potential sweep cycles between 0 and 1.0 V.

The signal dissipates only slowly after the potential is returned to and held at 0.0 V. Since the nonroughened (smooth) gold surface does not provide surface enhancement of Raman scattering, the enhancement of Raman bands of **ABTS**^+•^ is due to resonance enhancement of scattering from **ABTS**^+•^ only. Resonance enhancement is not a surface process, and therefore, the Raman signal originates from the whole (confocal) volume of solution at the working electrode (Figure 2). Although **ABTS**^+•^ is generated at the electrode surface, its concentration in the whole confocal volume increases over time only by diffusion from the electrode resulting in a lag in reaching the maximum intensity. However, a second process needs also to be considered: comproportionation of **ABTS** and **ABTS**^2+^.

**ABTS**^+•^ is formed by one-electron oxidation at the electrode already when the electrode is polarized to 0.5 V. The maximum intensity, however, is only obtained at a potential closer to 1.0 V. At 1.0 V, **ABTS**^2+^ is generated as well, but is not observed as its concentration in the Nernst diffusion layer cannot build up due to comproportionation with **ABTS** diffusing toward it from the bulk solution. Hence, **ABTS**^2+^ is formed at the electrode–solution interface, but is not present significantly in the Nernst diffusion layer.

This comproportionation, together with the time taken for the diffusion layer to develop, accounts for the lag between reaching sufficiently positive potentials for the one-electron oxidation of **ABTS** (i.e., 0.5 V) and the maximum Raman intensity of **ABTS**^+•^ obtained (at ca. 1.0 V). In fact, the observed delay corresponds closely to the time it takes to fill the confocal volume, calculated with typical diffusion coefficient in water for a small molecule (1 × 10^–5^ cm^2^ s^–1^), i.e., 1.25 s for 50 μm. Furthermore, the **ABTS**^+•^ in the Nernst diffusion layer and beyond is too far from the electrode surface to undergo reduction on the return cycles. Therefore, its concentration in the confocal volume of the Raman microscope, and by extension the observed Raman scattering, does not vary significantly on subsequent cycles. This delay means that, with a smooth gold electrode, processes in the Nernst diffusion layer are studied (i.e., comproportionation and diffusion), rather than processes immediately at the electrode surface.

### Raman Spectroelectrochemistry of ABTS at a Roughened Gold Electrode

**Figure 8:**
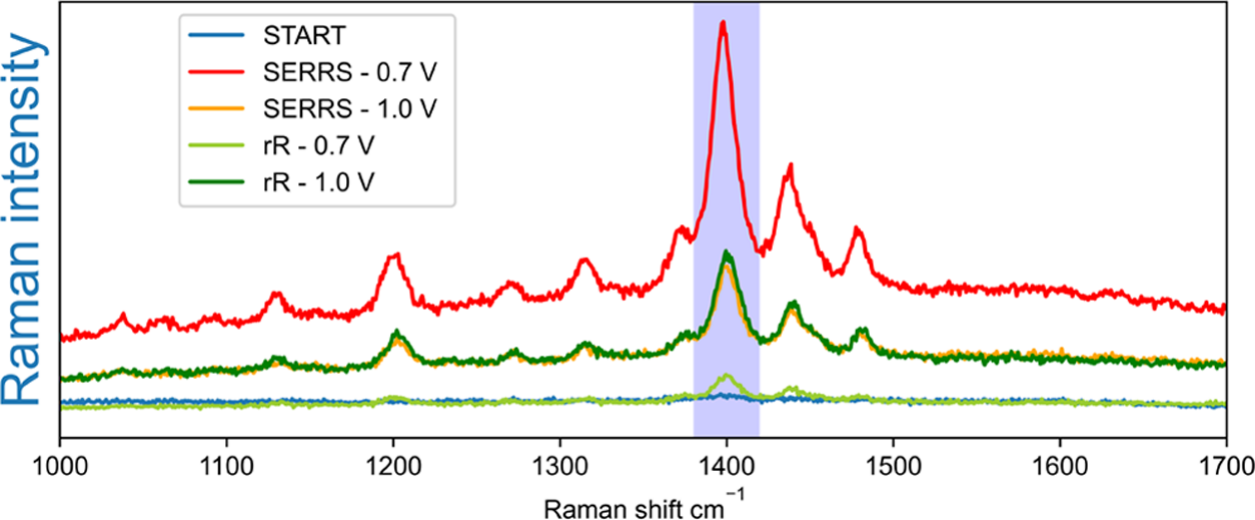
Resonance- and surface-enhanced resonance Raman spectra (λ_exc_ 785 nm) of **ABTS**^+•^ (0.65 mM) in water. Resonance Raman (rR) spectra were obtained using a smooth gold working electrode. Exposure time is 1 s. Surface-enhanced (SERRS) spectra were obtained with a roughened gold bead working electrode, each at different potentials during the first CV cycle. The area of the band at 1400 cm^–1^ (highlighted) was integrated for kinetic analysis (vide infra). Scan rate 0.1 Vs^–1^.

**Figure 9:**
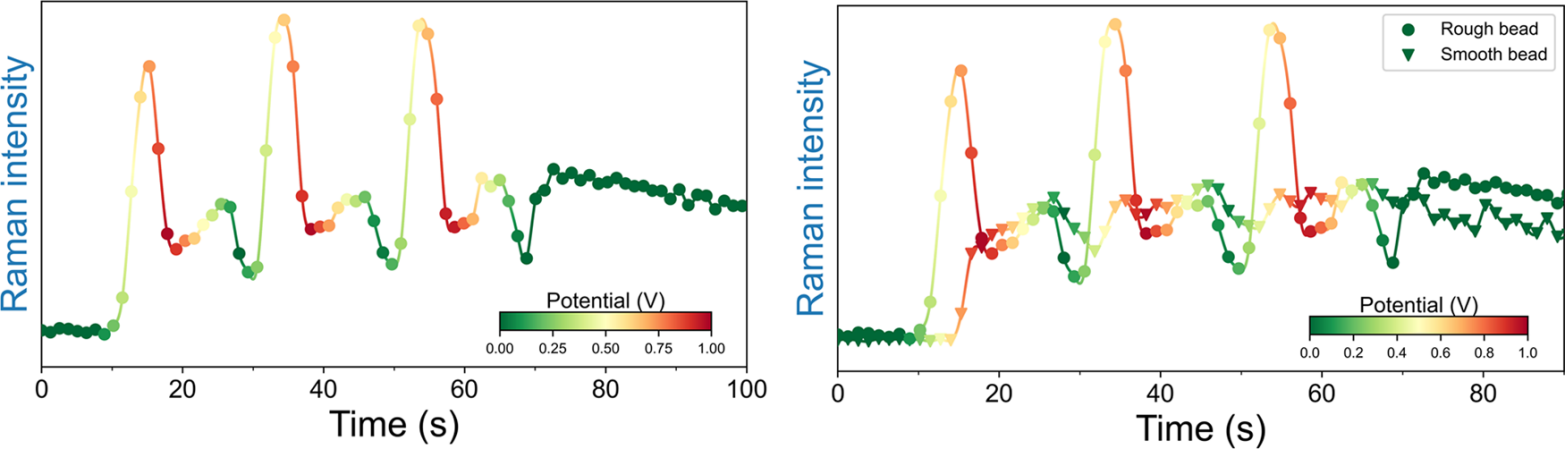
(Left) Surface-enhanced resonance Raman intensities of the band at 1400 cm^–1^ recorded at a roughened gold working electrode during cyclic voltammetry from spectra in Figure 8. (Right) Comparison between a roughened and smooth bead (see Figure 7). Minimum was 0.0 V (green) and maximum 1.0 V (red). Scan rate 0.1 Vs^–1^.

Cyclic voltammetry at a roughened (and hence SERS-active) gold electrode shows similar changes in the Raman spectrum obtained over multiple cycles (Figure 8) as observed with nonroughened electrodes discussed in the previous section (Figure 7). The main difference is a significantly higher Raman intensity during the part of the voltammetric cycle where the potential favors generation of **ABTS**^+•^ (i.e., between 0.4 and 1.0 V, Figure 9). It should be noted that the concentration of **ABTS** used (0.65 mM) corresponds to a surface coverage of 10^–13^ mol cm^–2^, in terms of the moles present within 1 nm of the electrode surface, the distance from the electrode surface for which surface enhancement of Raman scattering is expected. This surface coverage corresponds to less than 1 per cent of a full molecular monolayer. Although the number density present is low, in the absence of strong scatterers that are specifically adsorbed to the electrode, the SERS intensity is still sufficient to be detected with modern instruments. Even though a much smaller amount of material is enhanced by the surface, compared to the number of molecules present in the confocal volume (<5 μm), a more intense signal is observed compared to that with a nonroughened bead (Figure 7). Hence, enhancement by interaction with the surface plasmon is orders of magnitude more effective than resonance enhancement. It is of note that the normalized spectra at all potentials are identical, which is consistent with enhancement due to proximity to the surface and not due to adsorption of **ABTS** to gold.

A further difference with the nonroughened bead is that the Raman intensity during cyclic voltammetry with the roughened bead increases much more rapidly with increased electrode potential. Consequently, a peak intensity is observed at ca. 0.75 V, which is the midpoint between the first and second redox waves, at the point of maximum (Nernstian-dependent) concentration of **ABTS**^+•^ at the electrode. As the potential increases further to 1.0 V, the concentration of **ABTS**^+•^ at the surface decreases in favor of **ABTS**^2+^, demonstrated by the decrease in intensity of Raman bands of **ABTS**^+•^. On the return cycle, the concentration of **ABTS**^+•^ increases again as **ABTS**^2+^ still present at the electrode is reduced back to **ABTS**^+•^ at 0.8–0.3 V. This reduction leads to a smaller increase in Raman intensity, as the concentration of **ABTS**^2+^ available for reduction is reduced by comproportionation reactions and diffusion from the electrode.

The difference in the response time compared to the nonroughened bead is consistent with enhancement of Raman scattering by SERS for species close to the electrode (1–2 nm), which results in the immediate appearance of Raman bands of the radical cation once the potential is close to  of the first oxidation. As the potential is increased to that of the second redox wave (at 0.9 V, Figure 6), the concentration of **ABTS**^+•^ drops at the electrode, resulting in a loss of surface-enhanced Raman signal from the radical cation. However, since **ABTS**^2+^ diffuses away from the electrode and comproportionates with **ABTS** to form two equivalents of **ABTS**^+•^, a sufficiently high concentration of **ABTS**^+•^ is maintained within the confocal volume such that the resonance-enhanced bands of the latter are observed at 1.0 V. The resonance-enhanced bands are observed continuously, as was the case for measurements with the smooth bead (Figure 7). As the potential is swept negatively, an increase in **ABTS**^+•^ concentration at the electrode surface is followed by a decrease, and so the SERS enhancement is transiently observed to recover. When held at the final potential (0.0 V), the Raman intensity of the radical cation decreases steadily over time, as observed with the nonroughened electrode.

The surprisingly high intensity of the residual resonant bands, compared to the relative enhancement factors from the surface and due to resonance, is consistent with the difference in size of the SERS-active surface and the confocal volume (Figure 2). In solution, the total volume analyzed (>5 μm) is much larger than the volume at the surface (>1 nm) that is responsible for SERS. In other words, the fraction of molecules present at the surface close enough for SERS enhancement will be relatively small compared to the total number of molecules detected in the confocal volume, which is typically microns thick in solution.^13,28^

When working at concentrations that are too low even for resonance enhancement to provide detectable Raman signals (e.g., picomolar), surface enhancement can still be sufficient with strong scatterers such as aromatic dyes.^55,61^ Indeed, SERS spectra for **ABTS** present at such low concentrations have been reported earlier by Garcia-Leis et al.^60^ In the present study, we obtained Raman spectra during cyclic voltammetry at a roughened gold electrode and at such low concentrations also (Figure S22). In this case, the signal of **ABTS**^+•^ dissipates completely upon reversal of potential (Figure S23), as only the molecules at the surface benefit from surface enhancement and are, at the same time, close enough to the electrode to be reduced to the neutral state again.

### Raman Spectroelectrochemistry of TMA

**Figure 10:**
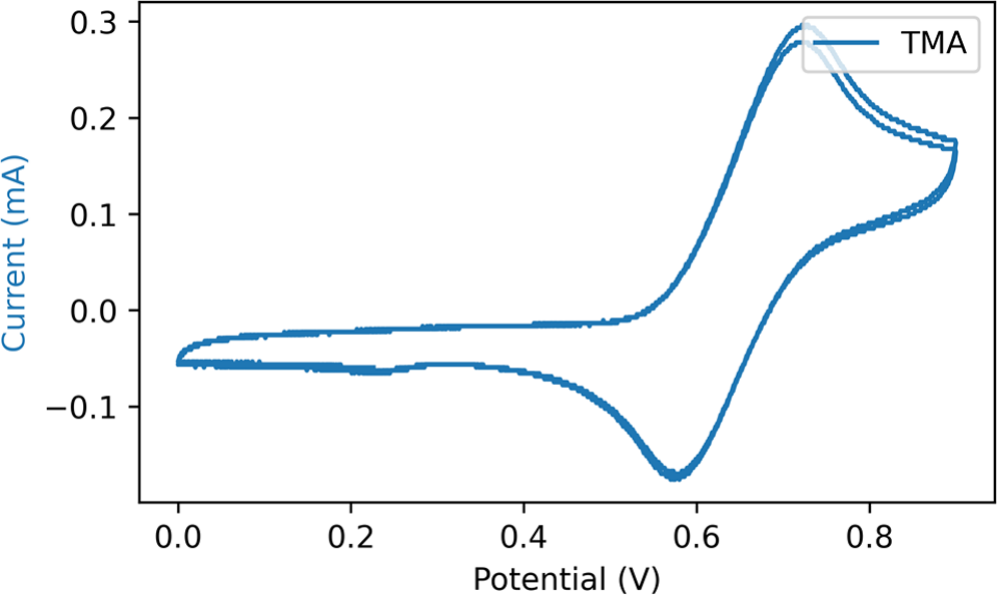
Cyclic voltammetry of **TMA** (1 mM) in CH_3_CN (0.1 M TBAPF_6_). Roughened gold working, Pt counter, and Ag/AgCl reference electrodes. Scan rate is 0.5 Vs^–1^.

Although resonance enhancement is useful when combined with SERS to study electrode reactions, typically, compounds of interest do not absorb in the same wavelength range in which surface-enhancement measurements are carried out (600–800 nm). A compound from the aniline family was chosen as an example of a nonresonant analyte, as it has been the focus of several recent studies on gold electrodes, including SERS.^23,41−43,62^ 4, *N*,*N*-Trimethylaniline (**TMA**) and its protonated (**TMA**–**H**^+^) and oxidized forms (**TMA**^+•^) do not show resonance enhancement at 785 nm. The methyl substituent at the para-position precludes radical dimerization/polymerization reactions from occurring upon electrochemical oxidation. The latter aspect is important as such coupling reactions produce species, such as *N,N,N’,N’*-tetramethylbenzidine or polyaniline (PANI)-type polymers, which adhere to the electrode and form highly colored cation radicals with strong resonant enhancement at 785 nm.^23,30^ For **TMA**, the methyl substituents largely block polymerization, and therefore, reversible one-electron oxidation is observed by cyclic voltammetry (Figure 10). The reversible redox behavior and lack of polymerization allows for analysis of periodic changes during cyclic voltammetry solely by SERS of species in solution.

**Figure 11:**
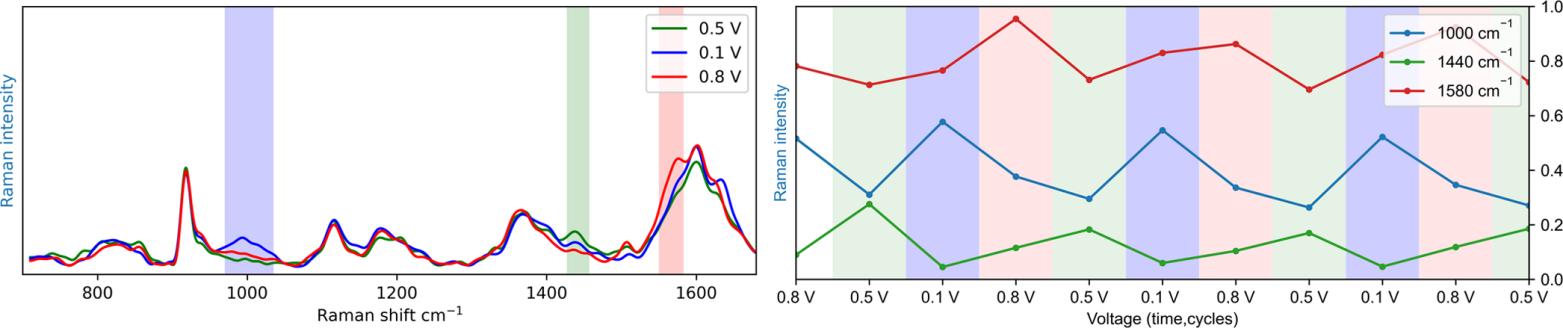
(Left) SERS spectra (λ_exc_ 785 nm) of **TMA** (1 mM) in CH_3_CN (0.1 M TBAPF_6_) at various electrode potentials. Exposure time is 5 s. The potential was kept constant during spectral acquisition. (Right) Integrated areas of the bands at 1000 cm^–1^ (blue), 1440 cm^–1^ (green), and 1580 cm^–1^ (red), with respect to potential.

The SERS spectra of **TMA** recorded during cyclic voltammetry, using a roughened gold bead as a working electrode, showed contributions from various redox states of **TMA**, with several Raman bands appearing and disappearing periodically. This periodicity means it is likely that these bands are associated to the distribution of species at each potential. Spectra recorded at 0.1, 0.5, and 0.8 V vs Ag/AgCl are shown in Figure 11(left). Although two species were expected, (**TMA** and **TMA**^+•^), the data indicated the transient presence of a third species.

The observed bands were found to be in good agreement with the hypothesis that **TMA** exhibits three distinct states:

- 1000 cm^–1^: N–H stretch (broad)^23,41,43^ of **TMA**–**H**^+^
- 1445 cm^–1^: C–H deform, ring stretch (ring, benzenoid)^41,42^ of **TMA**
- 1580 cm^–1^: breath (ring, para-oxidized aniline)^41,42^ of **TMA**^+•^

**Scheme 2:**
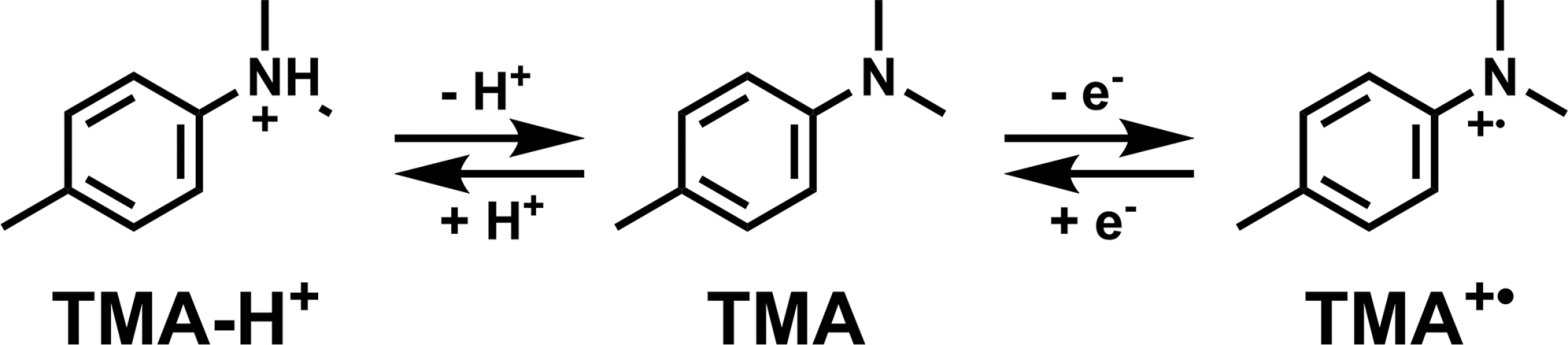
Proposed Structural Changes of **TMA** at 0.2, 0.5, and 0.8 V from Left to Right

These observations are in line with earlier studies on aniline and similar compounds^23,41−43,55,62,63^ and are consistent with the redox pair observed in the cyclic voltammogram of **TMA** (Figure 10) and protonated **TMA** (**TMA**–**H**^+^) at 0.1 V vs Ag/AgCl, which has also been observed as an intermediate in **TMA** dimerization.^63^ Earlier studies have shown that the proton concentration at the electrode can increase considerably when high positive potentials are applied,^11^ leading to significant effects on local pH. The generation of protons locally will lead to protonation of **TMA** (Scheme 2), resulting in a broad band at 1000 cm^–1^ in the Raman spectrum.^23,41^ Indeed, control measurements of **TMA** in acidic media confirm the appearance of this band (Figures S24 and S25). However, proton generation occurs at potentials where water is oxidized, ca. 1.0 V vs Ag/AgCl, and not at 0.1 V vs Ag/AgCl, the potential at which the Raman bands assigned to **TMA**–**H**^+^ are observed. The protons generated at higher potential are still present when the potential of the electrode is reversed. After **TMA**^+•^ is reduced, these protons protonate the neutral **TMA**. This hypothesis is supported by the observation that the band at 1000 cm^–1^ only arises after the first cycle (Figure S25).

As a control experiment for specific adsorption, a roughened gold bead was immersed in a solution of **TMA** (1 mM) in CH_3_CN. After two hours, the bead was removed and placed into **TMA**-free CH_3_CN, after which Raman spectra were recorded at λ_exc_ = 785 nm while the optical system was focused on the surface of the bead. The optimal focus was found using the decrease of Raman signal of acetonitrile. Raman bands of **TMA** were not observed. Since the absence of a SERS signal in itself is not a definitive demonstration of the lack of adsorption, the SERS activity of the surface was verified by subsequent immersion of the bead in a 10 μM solution of [Ru(bpy)_3_]^2+^ from which a SERS spectrum was readily recorded (see Figure 5, vide supra).

In addition, it should be noted that although adsorption does not interfere in this example, the initial and generated species are not necessarily equally soluble and deposition on the electrode can occur during voltammetry. Of course, the lower concentrations required for SERS, compared to nonresonant Raman spectroscopy, also reduce the extent of analyte adsorption.

## Conclusions

Electrochemically roughened gold electrodes serve as versatile substrates to study electrochemically driven processes over a wide potential range with Raman spectroscopy. The surface enhancement of Raman scattering provided by the surface plasmon of the roughened electrodes allows for real-time determination of the species present at the electrode–solution interface (i.e., within 1–2 nm of the surface). Concentrations well below the limit of detection for nonresonant Raman spectroscopy in bulk solution were analyzed. For some species, e.g., **ABTS**^+•^, resonance enhancement results in Raman scattering from the entire confocal volume of the Raman microscope. In this case, a combination of measurements using either a smooth or a roughened (SERS-active) electrode allows for distinguishing between species within a few nm of the electrode and those in the region of overlap between the Nernst diffusion layer and the confocal volume. SERS spectroscopy at electrodes is typically applied to SAMs and polymers that are immobilized on the gold surface. Here, we applied the technique without SAM immobilization to study redox processes at the electrode surface in real time, where the time-resolved nature of the experiments in particular yielded information on transiently present species, i.e., **TMA–H**^+^. The redox systems that can be studied are ultimately limited by the potential window in which the roughened gold electrode is stable, i.e., between ca. 0.2 and 1.1 V vs Ag/AgCl. We anticipate that this approach will find use in mechanistic studies of electric conversions in organic chemistry and electrocatalytic reactions.

## Supporting Information Available

Electrochemical surface-enhanced resonance Raman spectroscopy; preparation of roughened gold electrodes; sweep-step voltammetry cycles used during roughening of the gold beads; SEM image of a roughened gold electrode; SEM image of smooth gold bead; Raman spectra of ferrocene and ferrocenium; concentration dependence of Raman spectra of ferrocene in acetonitrile; extra bands observed during SERS spectroelectrochemical measurements on ferrocene; intensity of the Raman band of ferrocene at 1105 cm; oscillations in the area of the Raman band of Fc+ at 1113 cm; cyclic voltammograms of [Os(bpy)](PF6); cyclic voltammograms of roughened and smooth gold beads; UV–vis absorption spectra of ABTS; resonance Raman spectra at 785 nm of ABTS; Raman spectra of ABTS+•, TMA, and H2SO4; integrated intensity of the Raman band at 1400 cm (PDF)
