# Supplementary material for: Selective Analysis of Redox Processes at the Electrode Interface with Time-Resolved Raman Spectroscopy
Source: Langmuir. 2023 Jul 21;39(30):10383–94. doi: 10.1021/acs.langmuir.3c00633 (PMC10399290; doi:10.1021/acs.langmuir.3c00633)
Supplement: Supplementary file 1 — la3c00633_si_001.pdf [file la3c00633_si_001.pdf]

Supporting Information

Selective analysis of redox processes at the electrode interface with time-resolved Raman spectroscopy

W.J. Niels Klement,[†,‡] Jorn D. Steen,[†,¶] and Wesley R. Browne[*,†]

†*Molecular Inorganic Chemistry, Stratingh Institute for Chemistry, Faculty of Science and Engineering, University of Groningen, Nijenborgh 4, 9747 AG, Groningen, The Netherlands*

‡*Pharmaceutical Analysis, Groningen Research Institute of Pharmacy, University of Groningen. Antonius Deusinglaan 1, 9700 AD, Groningen, The Netherlands*

¶*Present address: Ångström Laboratory, Department of Chemistry, Uppsala University, Lägerhyddsvägen 1, Uppsala 751 20, Sweden*

E-mail: w.r.browne@rug.nl

Table of Contents

1 Electrochemical surface enhanced resonance Raman spectroscopy (EC-SERS) S2

2 Preparation of Roughened Gold Electrodes S4

3 Additional Raman spectroscopic Data S8

1 Electrochemical surface enhanced resonance Raman spectroscopy (EC-SERS)

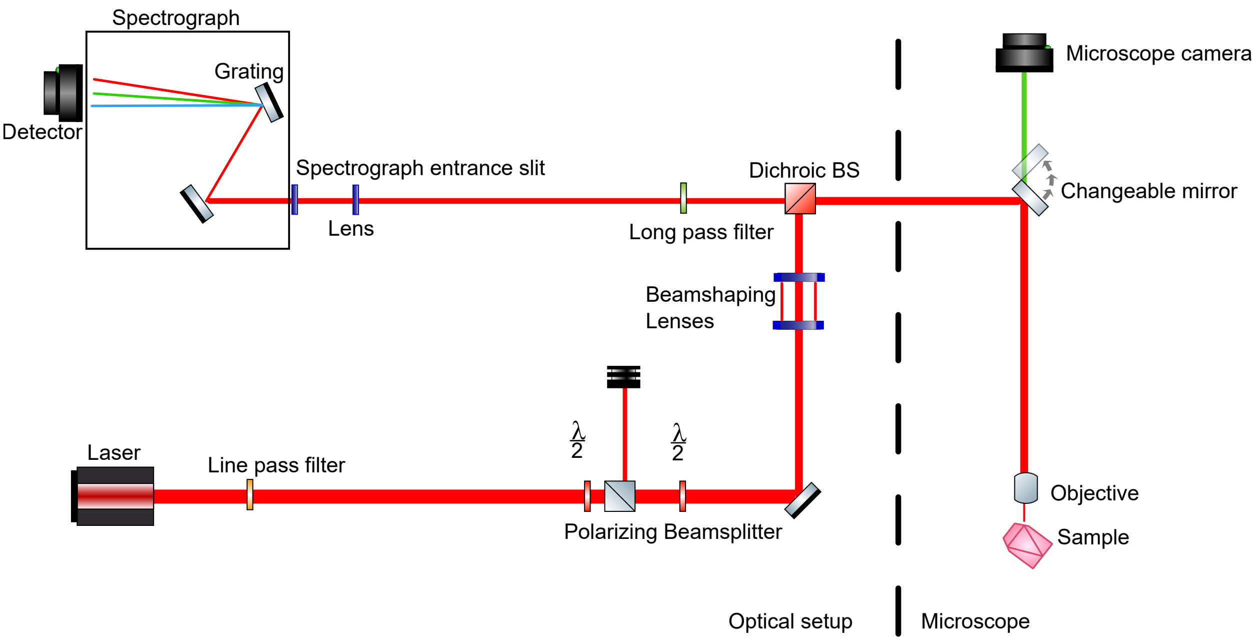

Figure S1: Optical arrangement used for Raman spectroelectrochemical measurements shown in Figure S2.

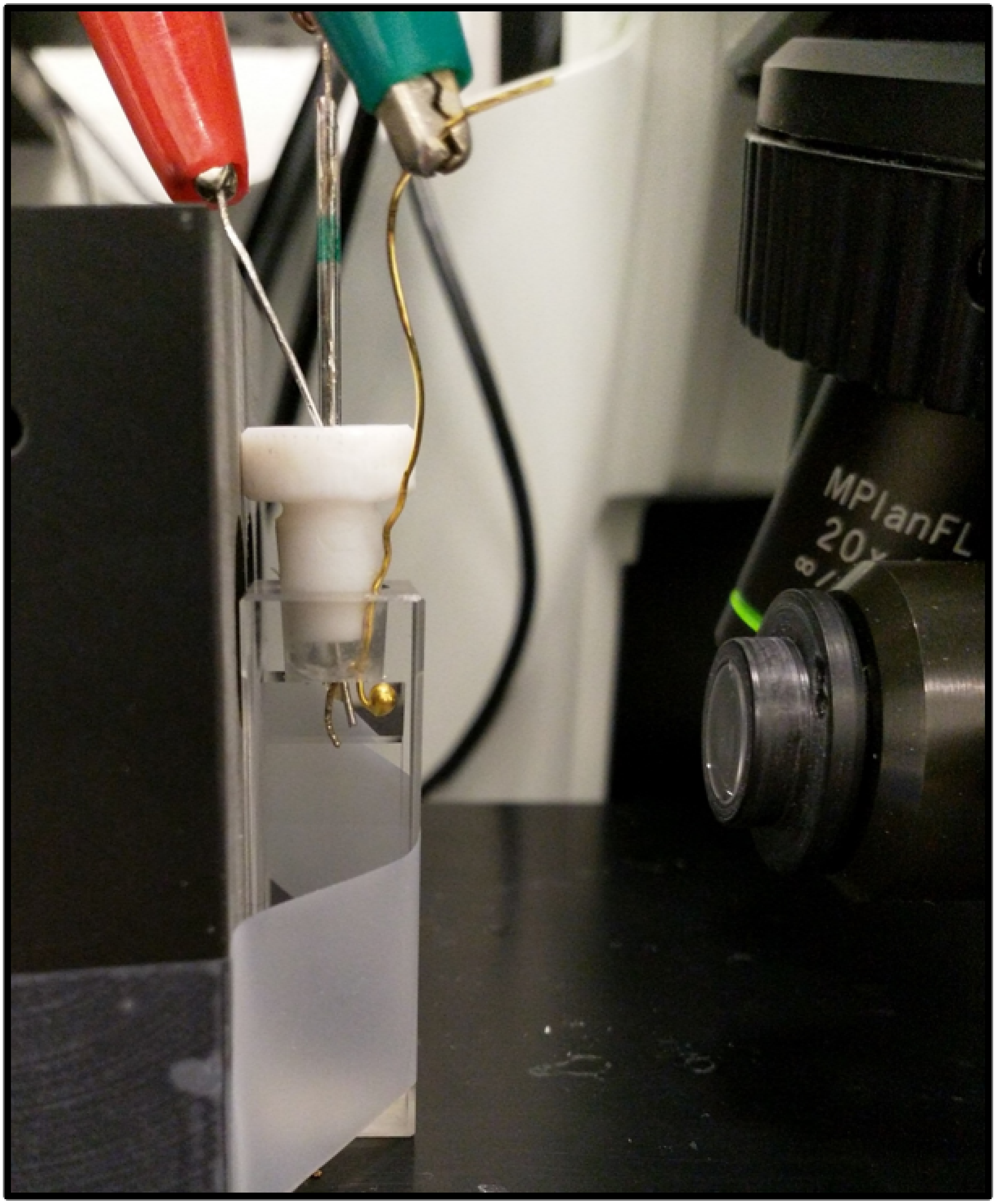

Figure S2: EC-SERS experiment. A BX51 microscope with periscope directed at the cuvette holding the electrodes on a X/Y/Z translation stage and electrical wiring to the potentiostat.

2 Preparation of Roughened Gold Electrodes

A gold bead was heated with a butane torch until it glowing orange. The bead was then immersed in aqueous sulfuric acid (0. 5 M) and held at 9 V with a Pt wire counter electrode until an orange coloration was observed. The bead was subsequently immersed in aqueous HCl (0.1 M) until it revert to a gold color. The bead was cycled between -0.6 V and 1.2 V for 12 cycles at 0.1 V/s in aqueous sulfuric acid (0.5 M) with a platinum wire counter electrode and Hg/$HgSO_4$ reference electrode. The electrode surface was roughened using a sweep step function. The bead was immersed in 0.1 M KCl(aq) with a Pt-wire counter electrode and SCE reference electrode. The potential was swept from -0.3 to 1.2 V at 1 V/s, held at 1.2 V for 30 s, swept back to -0.3 V at 0.5 V/s and held at -0.3 V for 3 s. This step was repeated 24 times for each bead. The first three cycles are shown in figure S3.

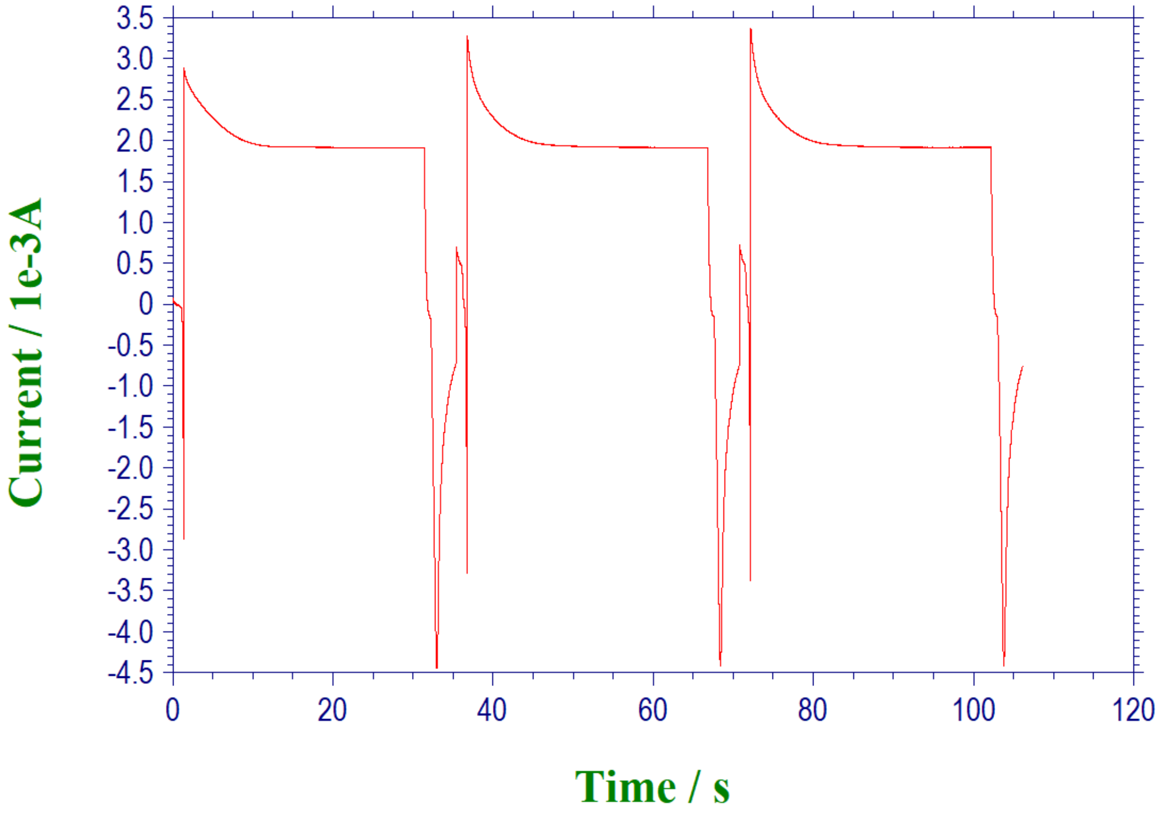

Figure S3: Sweep step voltammetry cycles used during roughening of the gold beads.

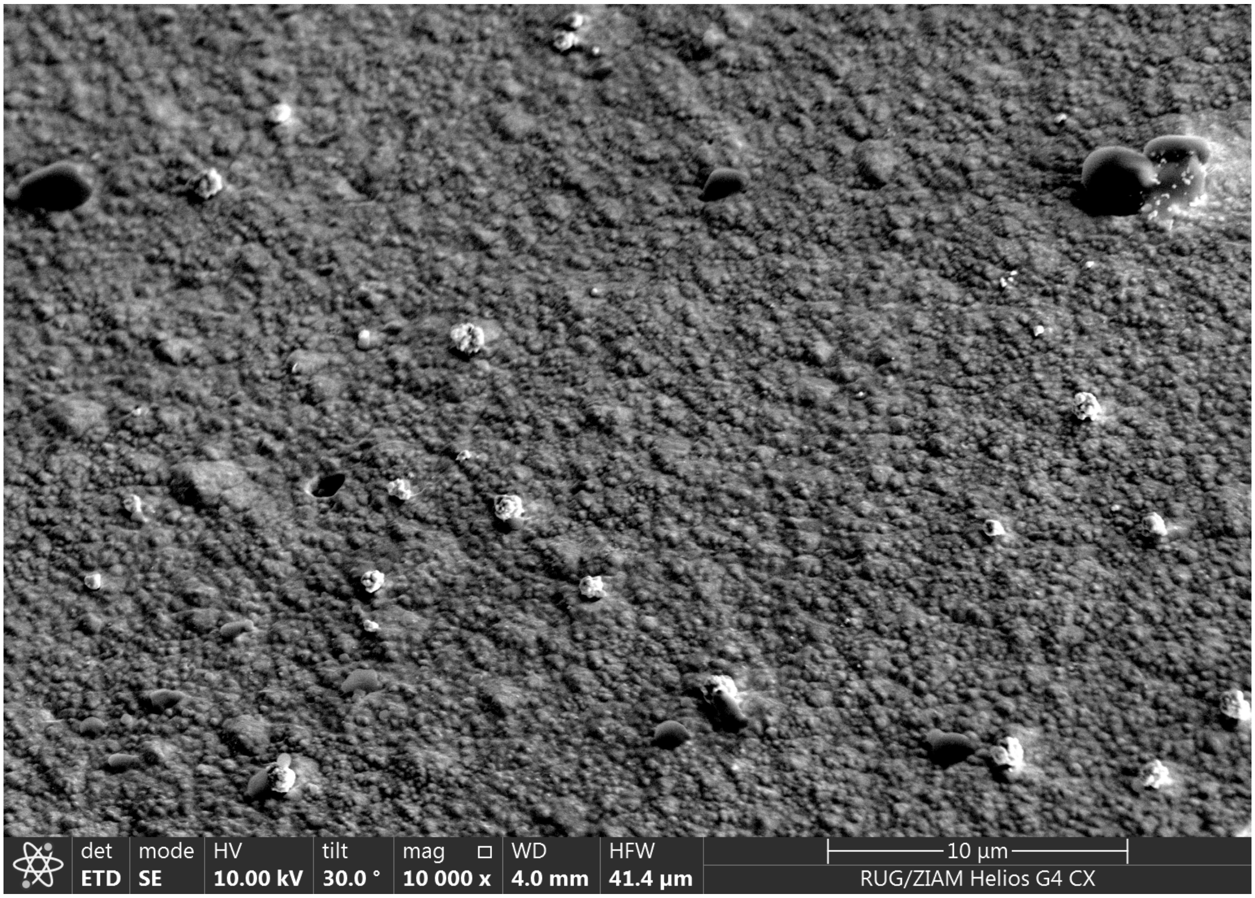

Figure S4: SEM image of a roughened gold electrode. Full image from the sub-image shown in Figure 2

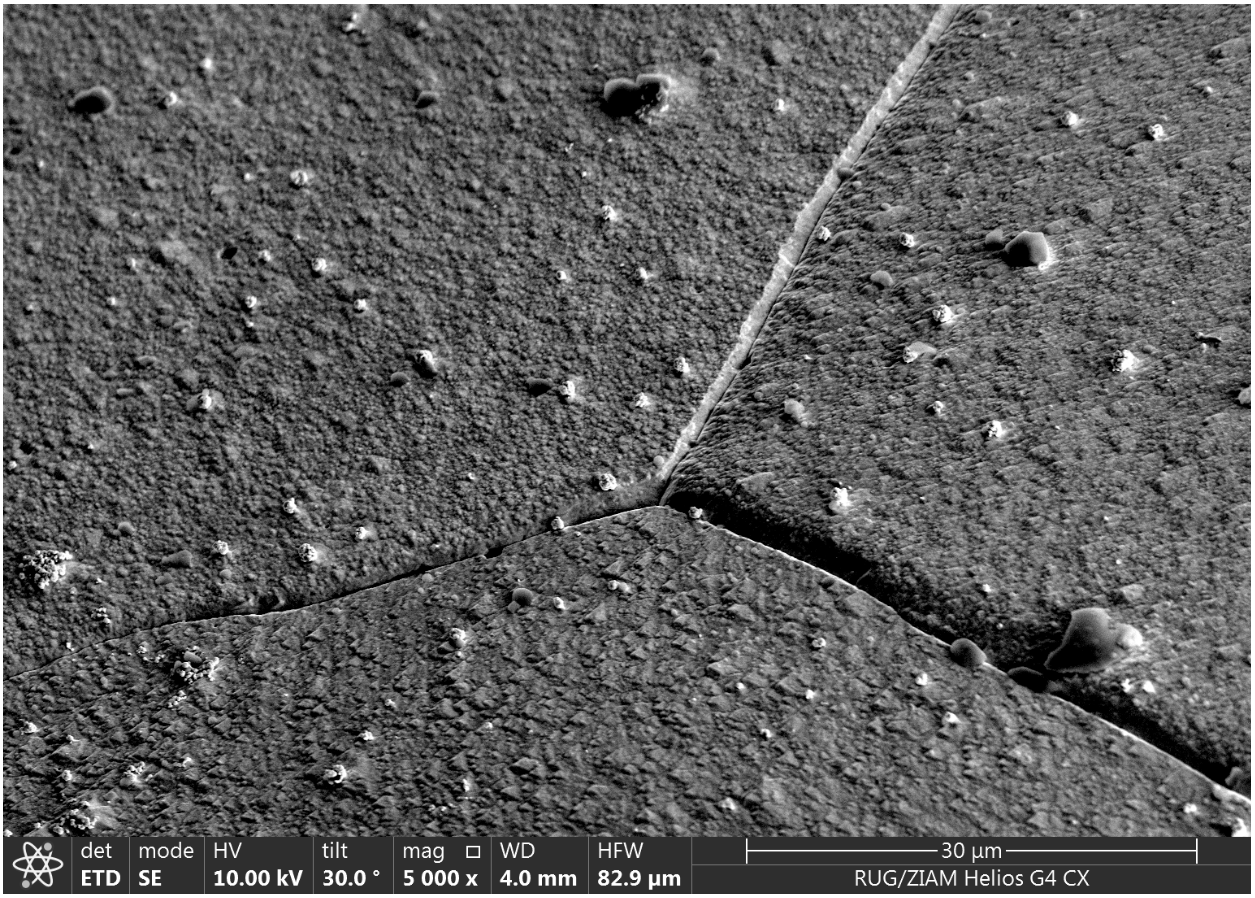

Figure S5: The surface of the roughened gold bead exhibit grain boundaries such as the triple grain boundary shown here. The nature of the surface roughness varies over the different grains and it is likely that it is this difference that is responsible for the point to point variation in SERS intensity obtained with this class of SERS substrate. Furthermore, this variation means that (potentio)dynamic studies carried out without change in the position of the bead can be quantitative whereas comparison between two measurements in which the position is changed cannot be compared absolutely, even for the same bead.

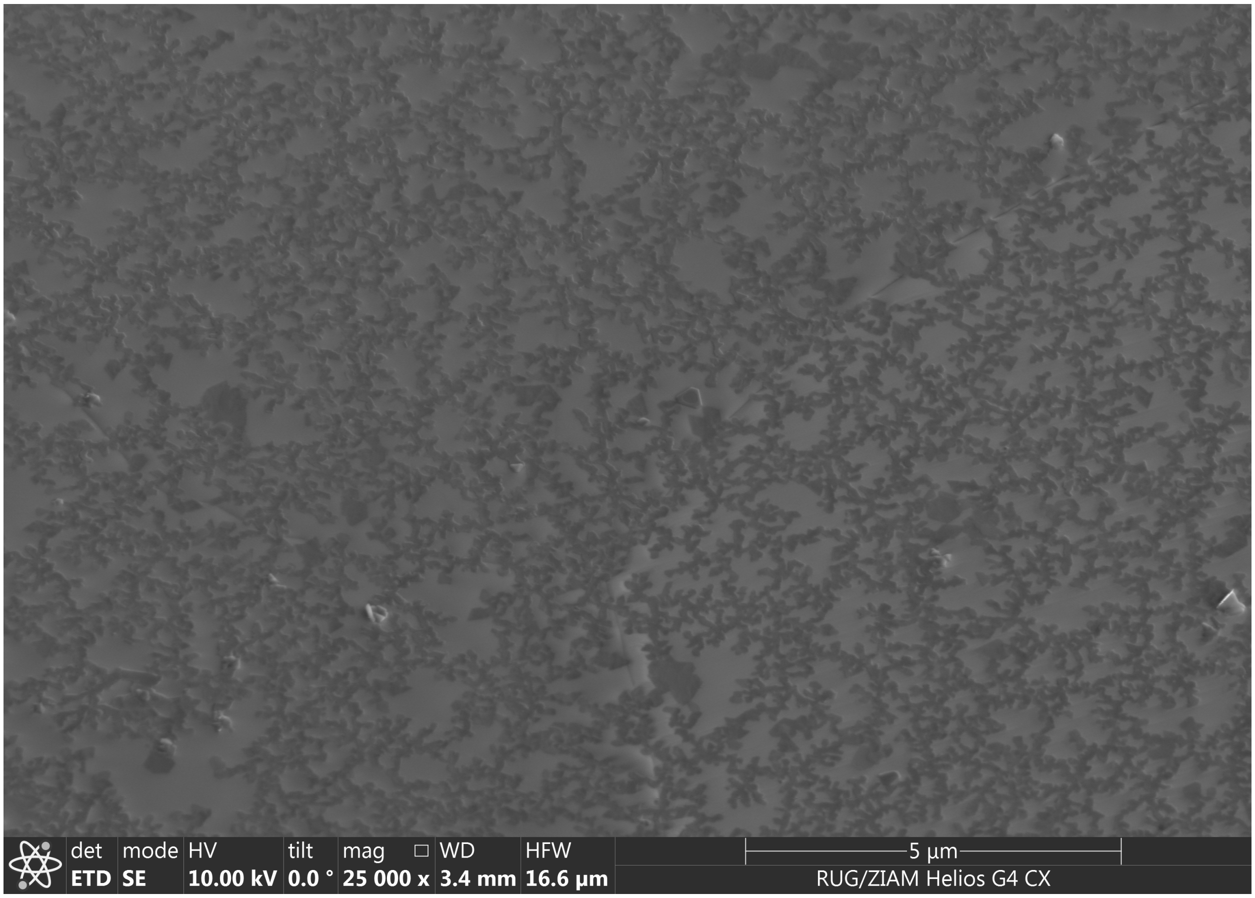

Figure S6: SEM image of smooth gold bead. Patterns are observed on the surface, but not roughness to a similar extent as the roughened surface, vide supra Figure S4. The patterning is likely caused by defect lines along the surface along with impurities in the gold that rise to the surface when the bead is heated to melt in the cleaning process. While surface defects can be useful in the roughening process (i.e. atoms are more easily oxidized off the surface near a defect), surface impurities are the reason an oxidative cleaning step is used in the roughening procedure.

3 Additional Raman spectroscopic Data

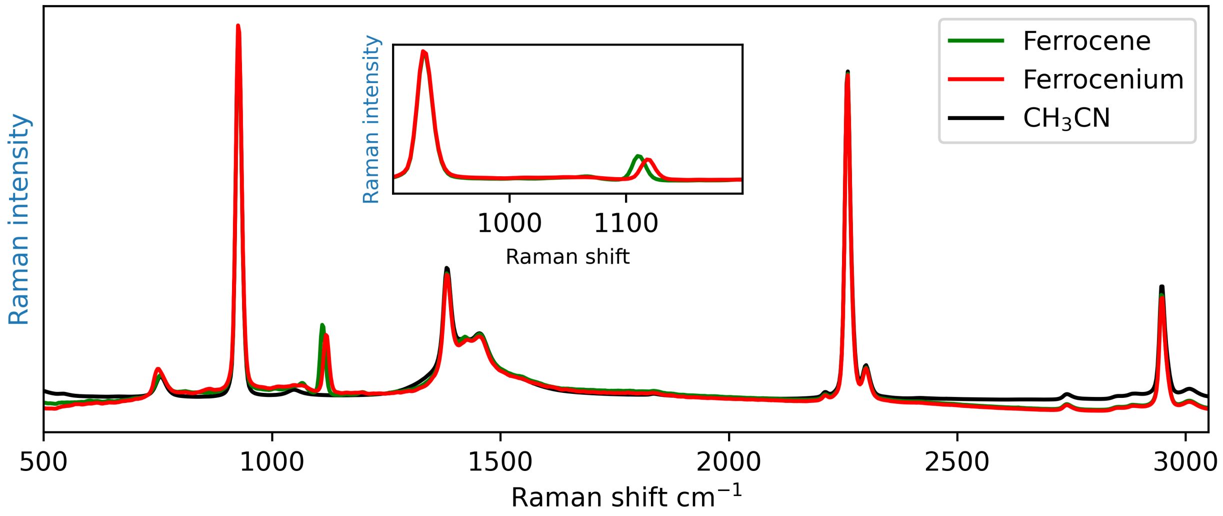

Figure S7: Raman spectra (λ_{exc}785 nm) of ferrocene (100 mM) (**Fc**), ferrocenium (100 mM) (**Fc**$^+$) in CH_3CN. Observed bands and relative intensity are similar to those obtained with surface enhancement. Inset: expansion of the same region as in Figure 3

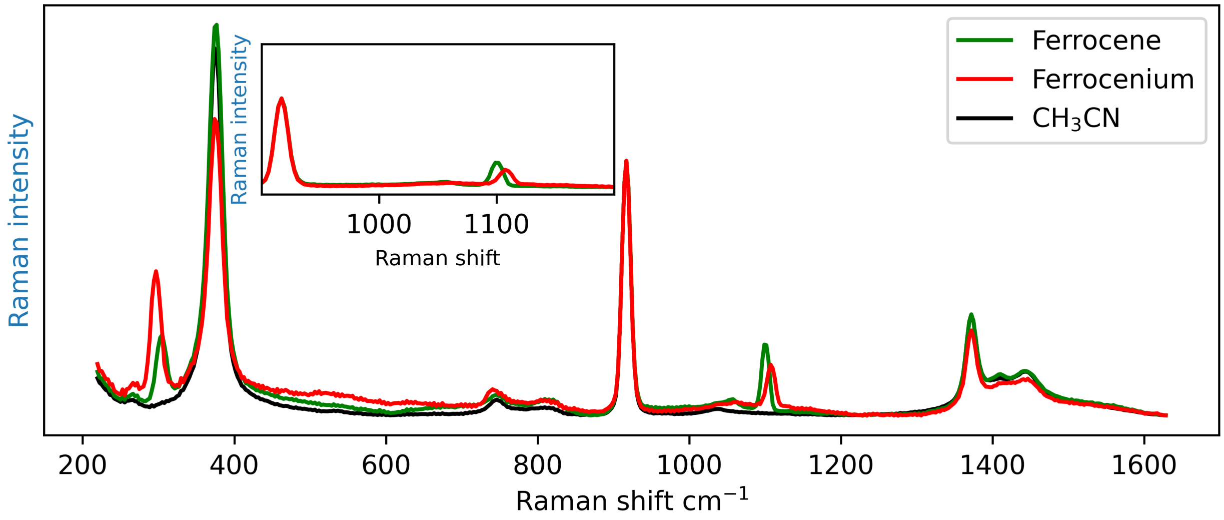

Figure S8: Raman spectra (λ_{exc}1064 nm) of ferrocene (100 mM) (**Fc**), ferrocenium (100 mM) (**Fc**$^+$) in CH_3CN. Observed bands and relative intensity are similar to those obtained with surface enhancement. Inset: expansion of the same region as in Figure 3

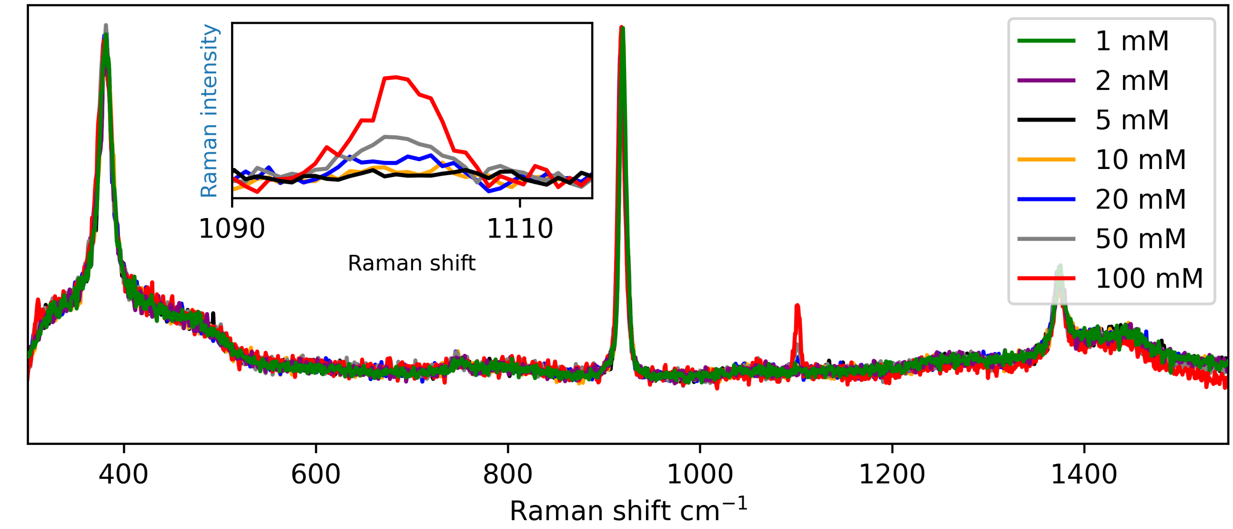

Figure S9: Concentration dependence of Raman spectra of ferrocene in acetonitrile. Exposure time 2 s, power at sample (785 nm) 3.3 mW. The detection limit of ferrocene was determined to be 20 mM with exposure time of 2 s and a laser power of 3 mW. A lower limit of detection can be obtained with longer acquisition times/high laser power, however, these spectral acquisition conditions were employed to compare to the SERS data directly. SERS measurements were carried out below 10 mM to exclude effects from non-resonant Raman scatter.

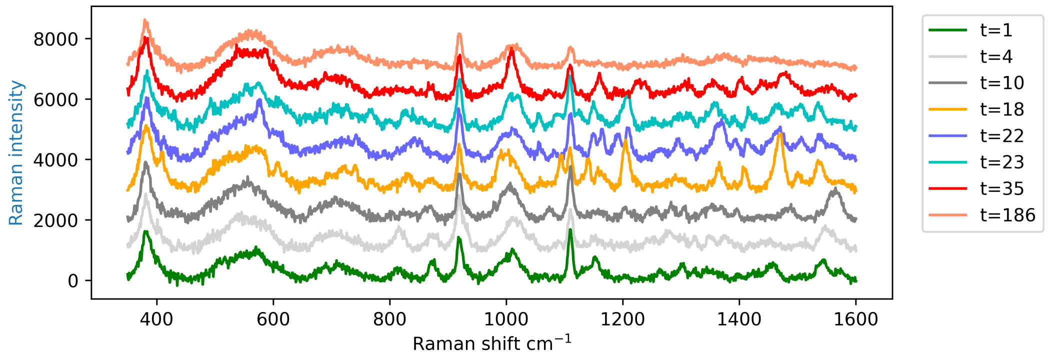

Figure S10: Examples of extra bands observed during SERS spectroelectrochemical measurements on ferrocene. The sporadic appearance of these bands is more frequent at the start of the measurement, and less after multiple cycles, in good agreement with expected oxidative surface cleaning. Averaging of multiple spectra or longer acquisition times result in a reduction in the relative contribution from these bands to the spectra, which may then appear as small bands and/or a less flat baseline, such as seen in Figure 3. Bands arising from the solvent and ferrocenium at 920 and 1113 cm^{-1} respectively, are present consistently throughout the experiment.

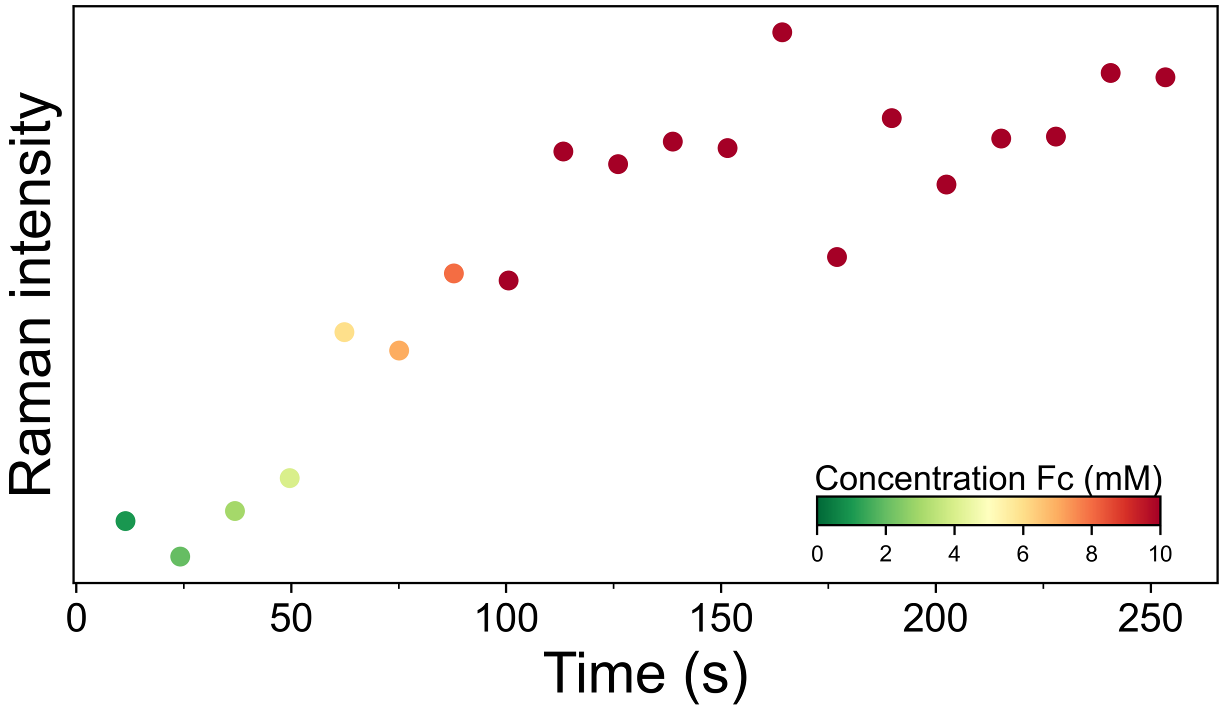

Figure S11: Intensity of the Raman band of ferrocene at 1105 cm^{-1} with increasing amounts added to the solution. Exposure time 2 s, power at sample (785 nm) 3.3 mW

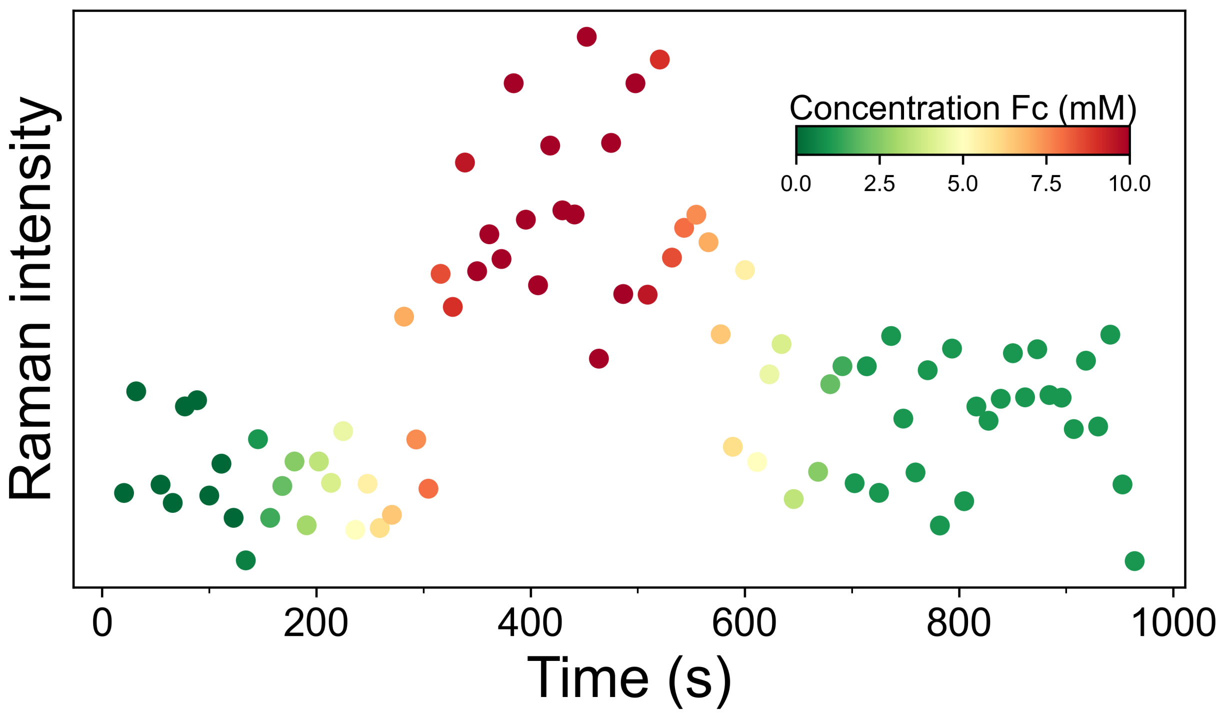

Figure S12: Intensity of Raman band of ferrocene at 1105 cm^{-1} with an increase in concentration followed by dilution. Exposure time 2 s, power at sample (785 nm) 3.3 mW.

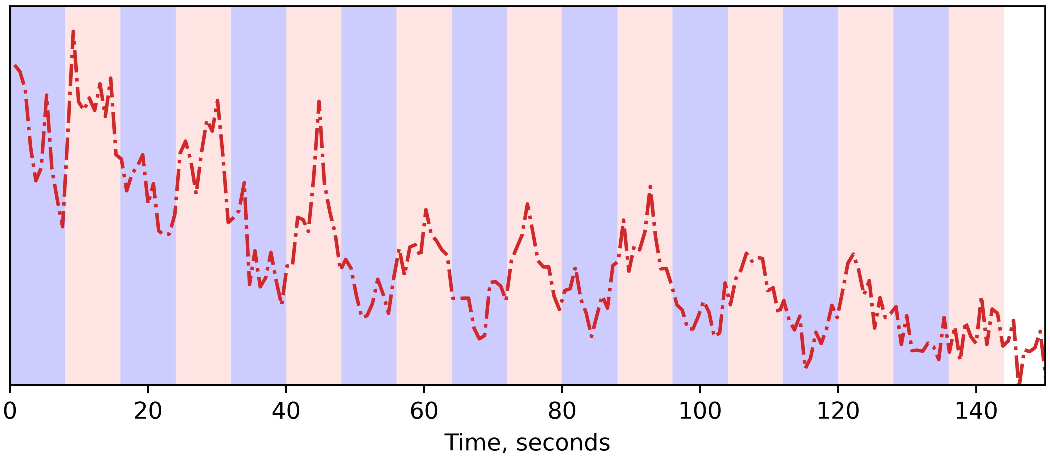

Figure S13: Oscillations in the area of the Raman band of $\mathbf{Fc}^{+}$ at 1113 cm^{-1}. The overall decrease in intensity is due to a decrease in contributions from adsorbed impurities over time and voltage cycles. Periods where the potential was swept positively are shown in red and negatively in blue highlights.

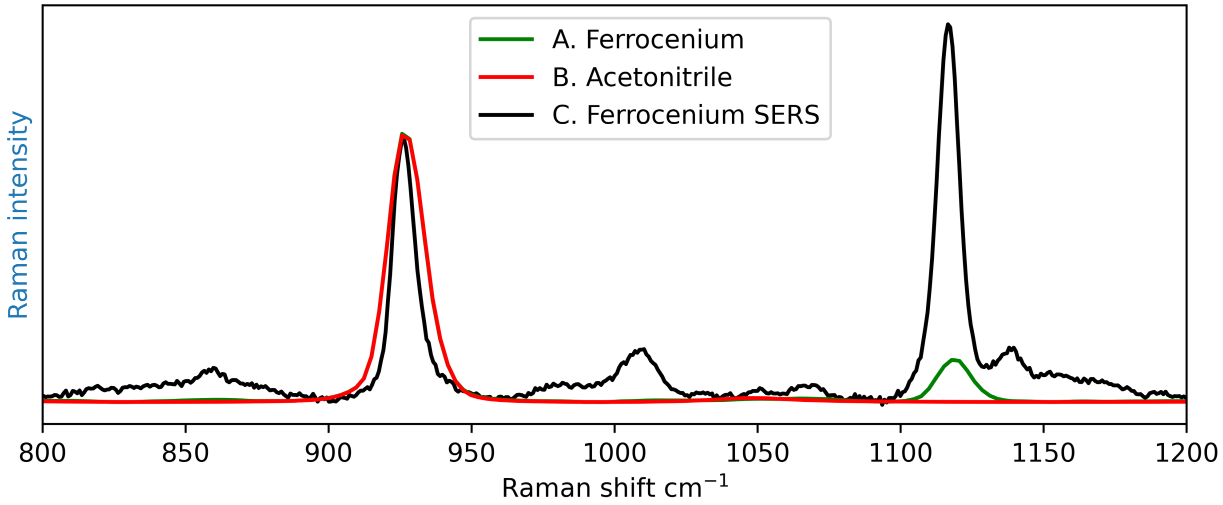

Figure S14: (A.) Raman spectrum (λ_{exc} 785 nm) of ferrocenium (100 mM) in CH_3CN, (B.) of CH_3CN, and (C.) Surface enhanced Raman spectrum of ferrocenium (1 mM) in CH_3CN. Spectra are normalized on the 919 cm^{-1} band of CH_3CN to highlight the relative difference in enhancement between the bands of CH_3CN and ferrocenium.

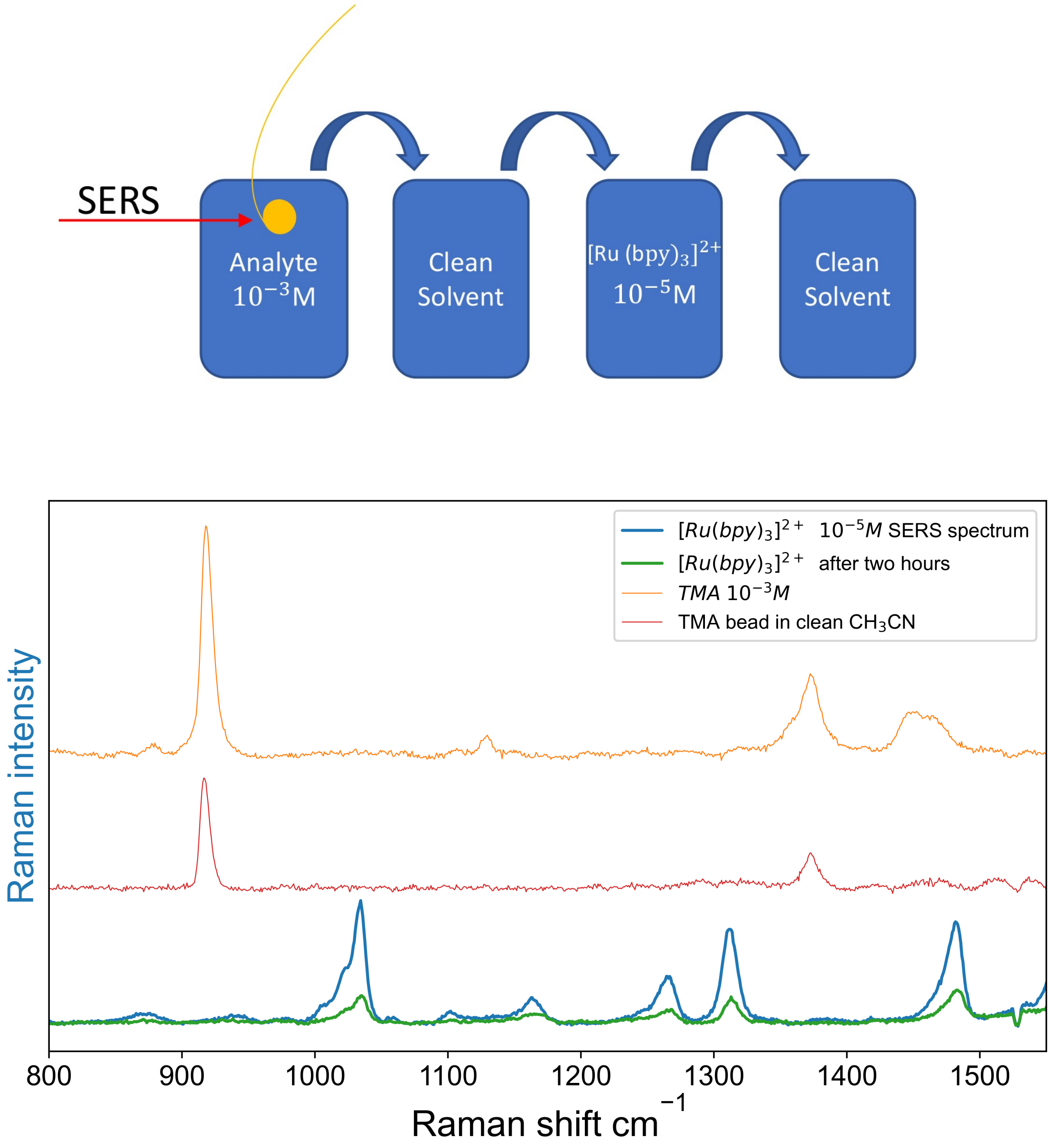

Figure S15: (top) Measurement sequence for adsorption test. A bead is first immersed in analyte solution (in this case **TMA**), followed by immersion in analyte free solvent. The bead is then immersed in a solution of $[Ru(bpy)_3]^{2+}$ as in Figure 5. (bottom) Raman spectra recorded at a roughened bead in a solution containing **TMA**, then in CH_3CN alone and in a solution of $[Ru(bpy)_3]^{2+}$.

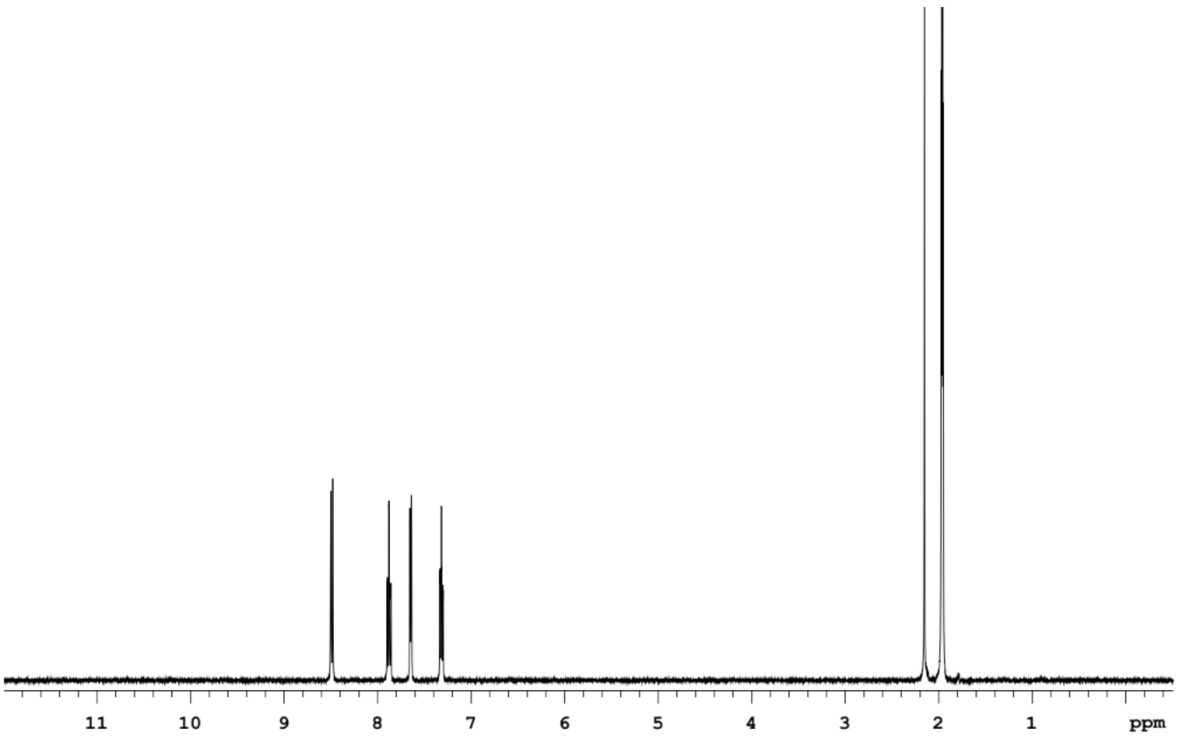

Figure S16: $[Os(bpy)_3](PF_6)_2$ was prepared by literature methods and characterized by ^1H NMR (400 MHz) spectroscopy in CD_3CN

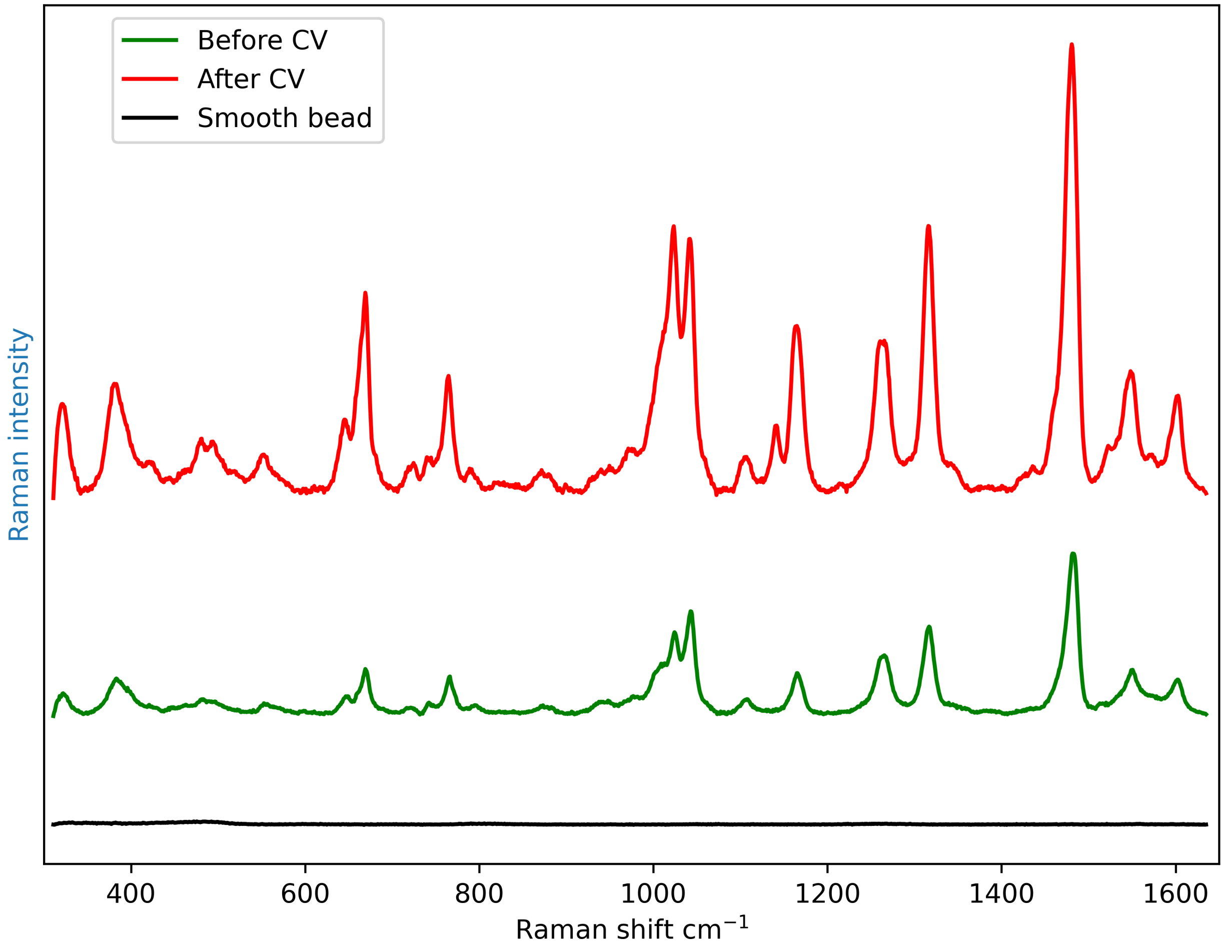

Figure S17: Raman spectra (785 nm, 3 mW at sample, 2 s exposure) of $[Os(bpy)_3](PF_6)_2$ adsorbed onto roughened and smooth gold beads (by immersion in a solution of $[Os(bpy)_3](PF_6)_2$ in CH_3CN were obtained after and rinsing with CH_3CN and drying) were recorded before and after cyclic voltammetry. The roughened gold bead showed the expected SERS spectrum of $[Os(bpy)_3](PF_6)_2$ whereas the bands of the complex could not be detected on the smooth gold bead.

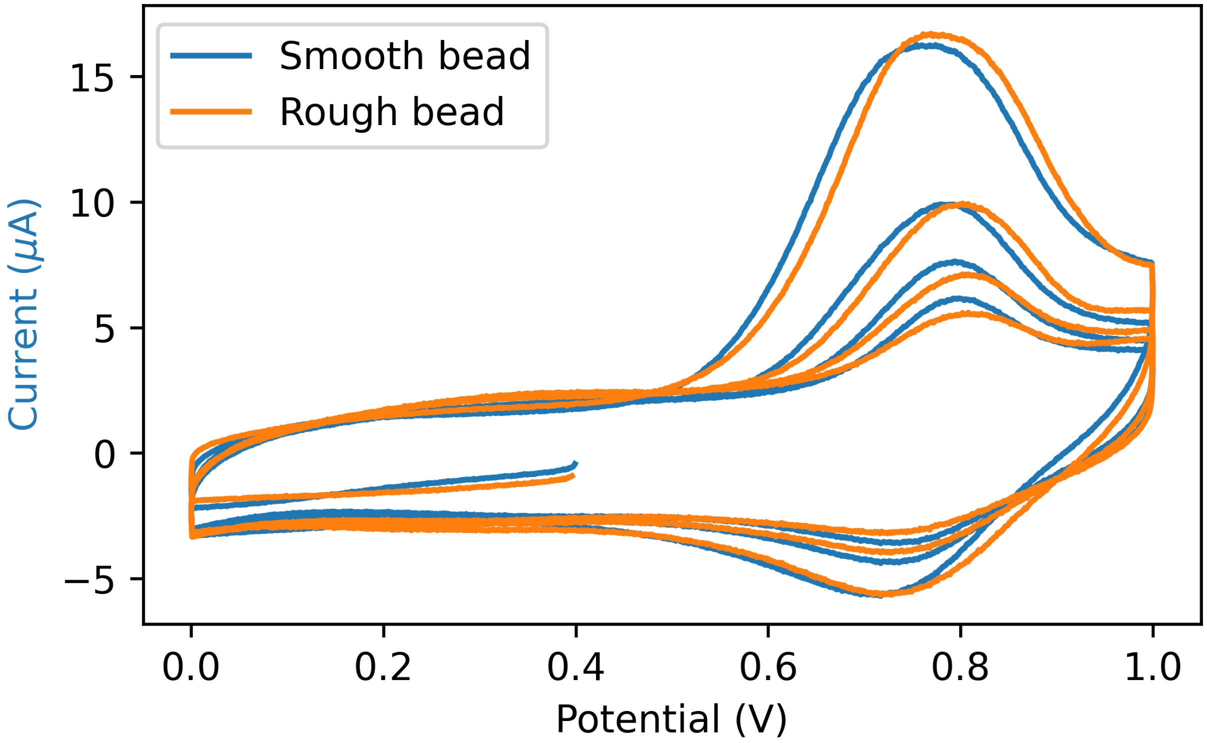

Figure S18: Cyclic voltammograms of $[Os(bpy)_3](PF_6)_2$ adsorbed on roughened and smooth gold beads. Scan rate is 1 V s^{-1}, vs Ag/AgCl in CH_3CN (0.1 M $TBAPF_6$).The anodic and cathodic currents for both beads were similar consistent with surface roughening having minimal impact on electrochemical surface area. The currents decreased on each cycle due to desorption caused by disturbance of the double layer during voltammetry. The surface density (Γ, mol cm^{-1}) of $[Os(bpy)_3](PF_6)_2$ on each electrode was determined from the charge passed on the first cycle, which was ca. 2.5 $\times 10^{-6}$C for both electrodes, corresponding to ca. 2.5×10^{-11} mol. The charge passed on the last cycle is 10% of the initial charge and hence the surface coverage is decreased to 10%.

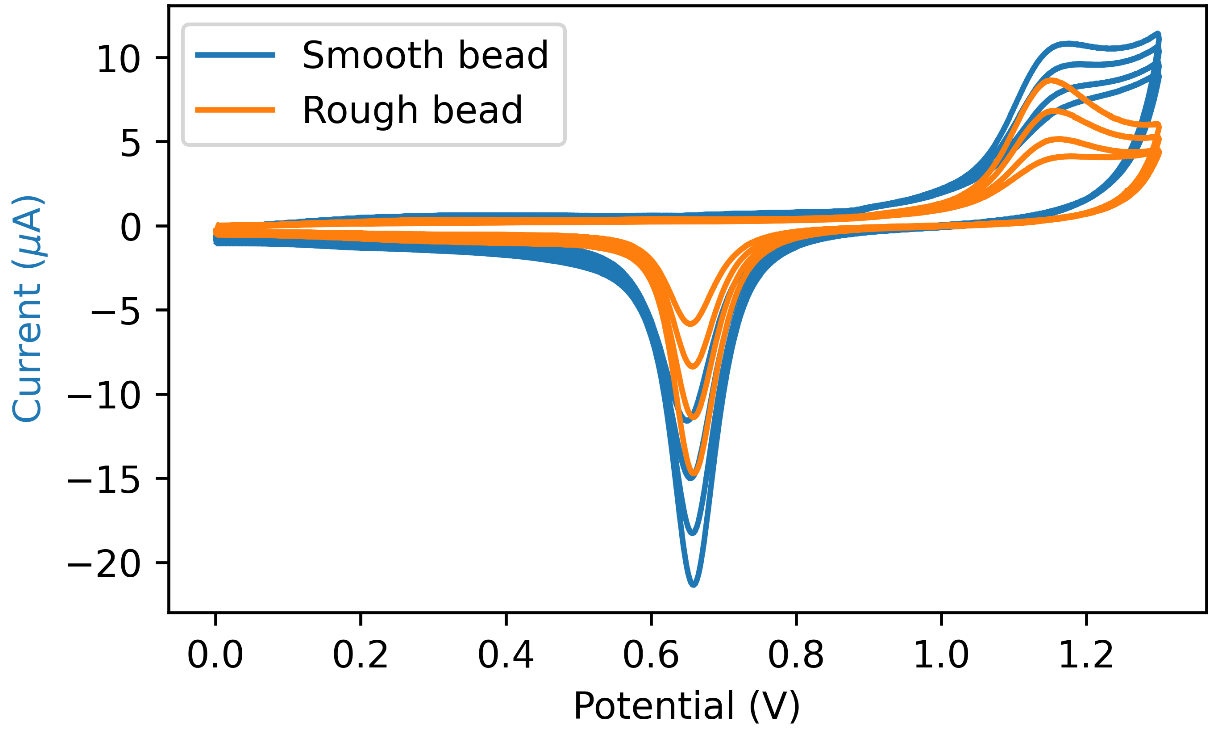

Figure S19: Cyclic voltammograms of roughened and smooth gold beads (see Figure S18 in 0.5 M H_2SO_4. Scan rate is 0.1 V s^{-1}, vs Ag/AgCl.The charge passed for the smooth bead was 1.1 to 1.5×10^{-5} C and for the rough bead 0.54 to 1.1×10^{-5} C. The electrochemical area was calculated using the relation $Q/A = 390$ μC cm^{-2}, to be 0.03 cm^{-2} and 0.028 cm^{-2}, respectively and hence a surface coverage of ca. 10^{-10} mol cm^{-2} corresponding to a full monolayer of $[Os(bpy)_3](PF_6)_2$ initially.

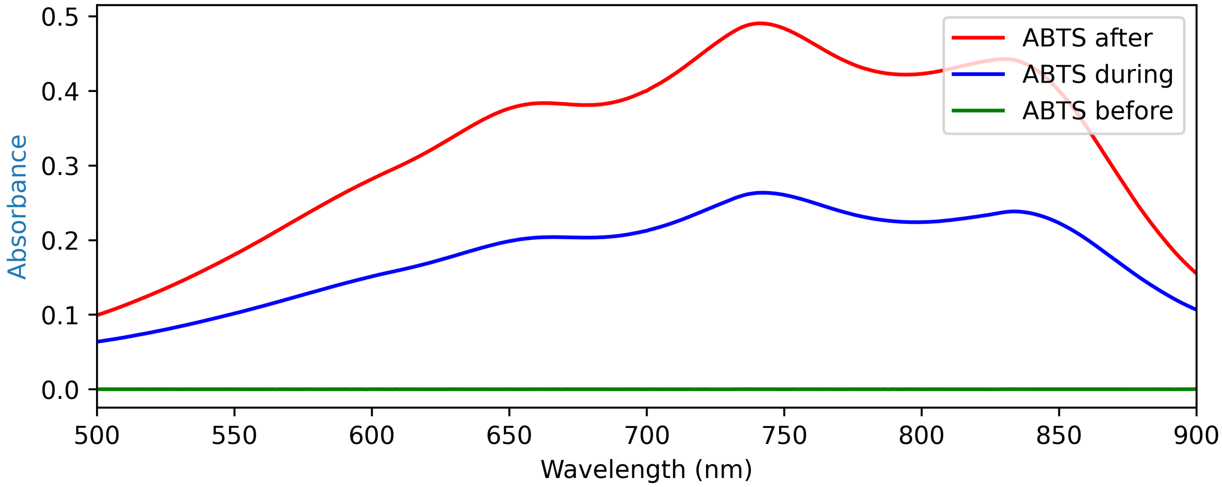

Figure S20: UV-Vis absorption spectra of **ABTS** (0.65 mM) in water before (green) during (blue) and after (red) oxidation by H_2O_2 (5 eq.) and catalyst $[Mn_2^{III}O_3(TMTACN)_2](PF_6)_2$ (0.5 eq.).

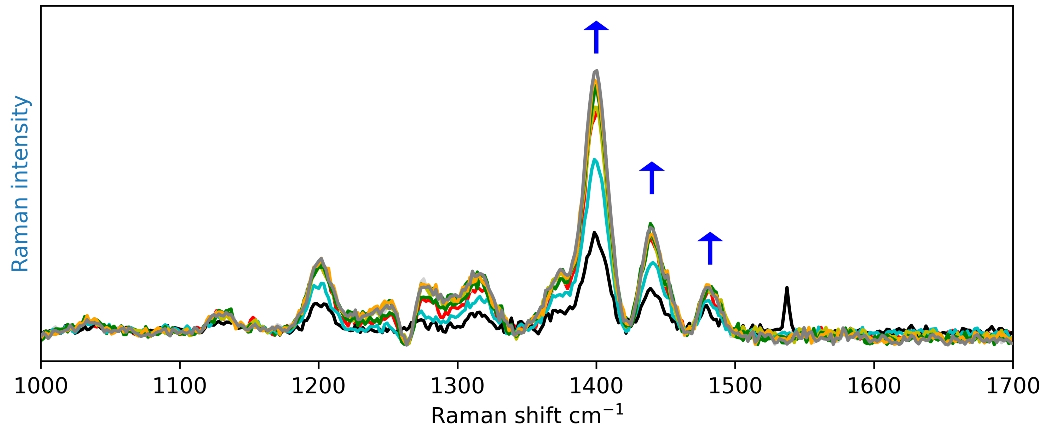

(a)

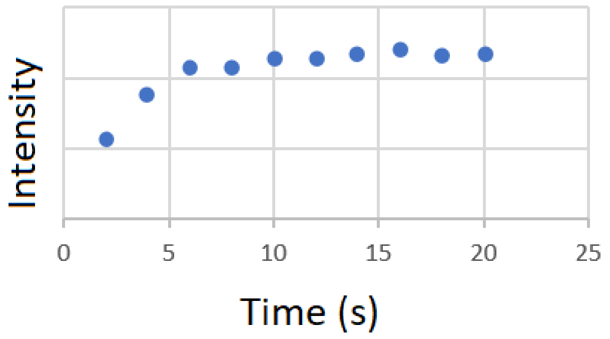

(b)

Figure S21: (a) Resonance Raman spectra at 785 nm of **ABTS** (0.65 mM) in water during oxidation with H_2O_2 (5 eq.) and the catalyst $[Mn^{III}_2O_3(TMTACN)_2](PF_6)_2$ (0.5 eq.). (b) Integrated Raman intensity at 1400 cm^{-1} over time

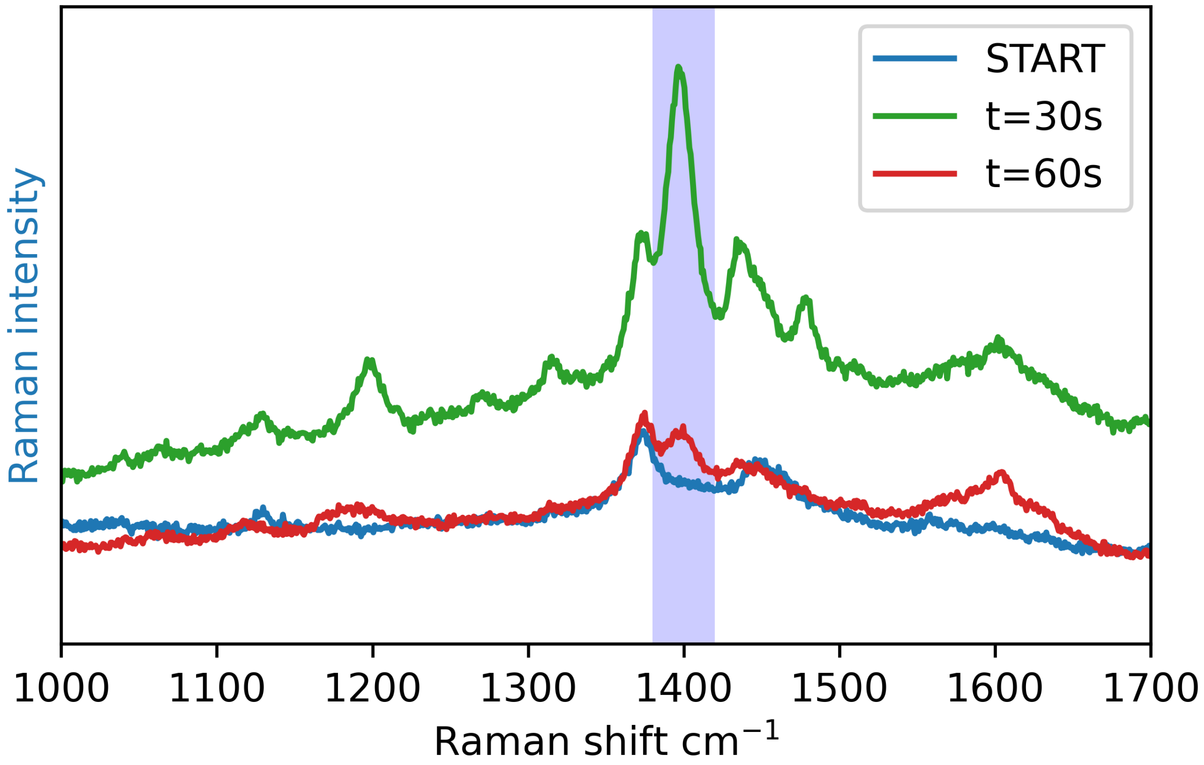

Figure S22: Raman spectra (λ_{exc} 785 nm) of **ABTS$^{+\bullet}$** (ca. 10^{-12} M) in water recorded during cyclic voltammetry with a roughened gold bead working electrode, with Pt counter and Ag/AgCl reference electrodes. Exposure time is 5 s.

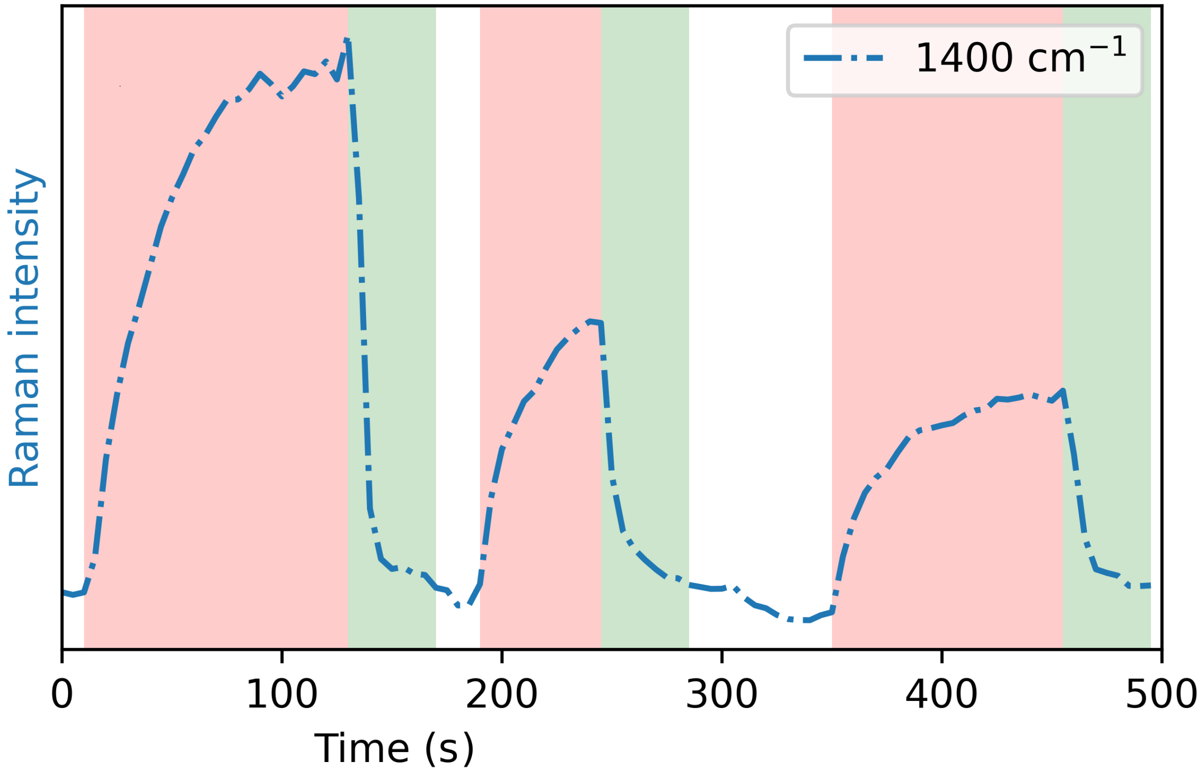

Figure S23: Integrated intensity of the Raman band at 1400 cm^{-1} of Figure S22. The concentration is too low for detection of **ABTS** by resonance Raman spectroscopy and hence only the SERRS bands are observable. In contrast to that shown in Figure 5, the observed signal decreases to the baseline when the potential is < 0.4 V. The potential was swept (from 0.0 to 1.0 V, at 10 mV/s and potential held constant at 1.0 V, shown in red, and at 0.0 V shown in green highlighted areas. Exposure time is 5 s.

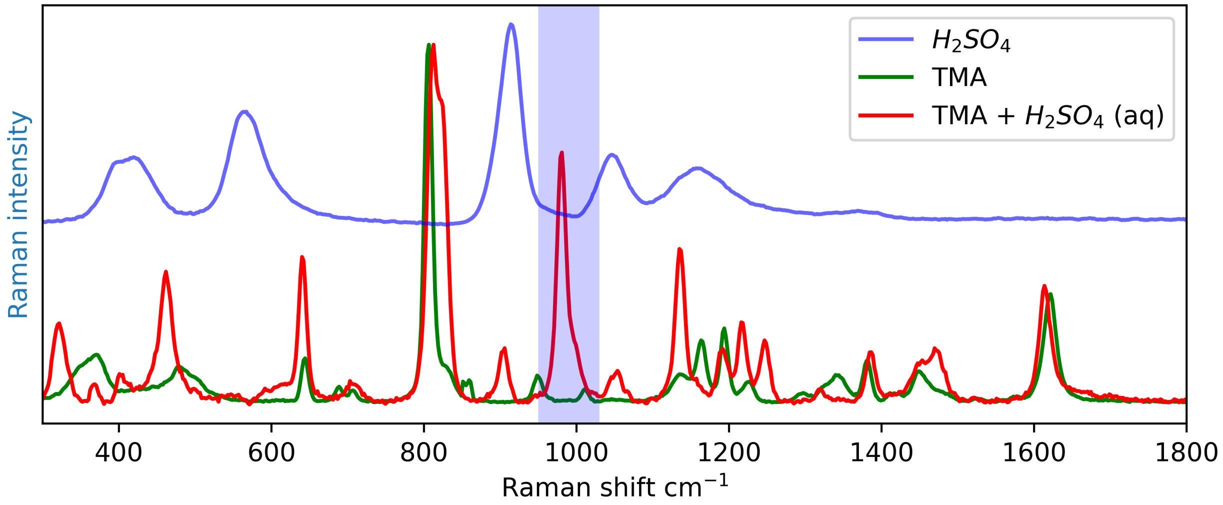

Figure S24: Raman spectra (λ_{exc} 785 nm) of H_2SO_4 (blue), neat **TMA** (green) and a 1:1:1 vol/vol mixture of **TMA**:H_2O:H_2SO_4 (red) yielding an intense, broad band at 1000 cm^{-1} (highlighted, assigned to the N-H stretch of **TMA−H$^+$**), see also Figure 9.

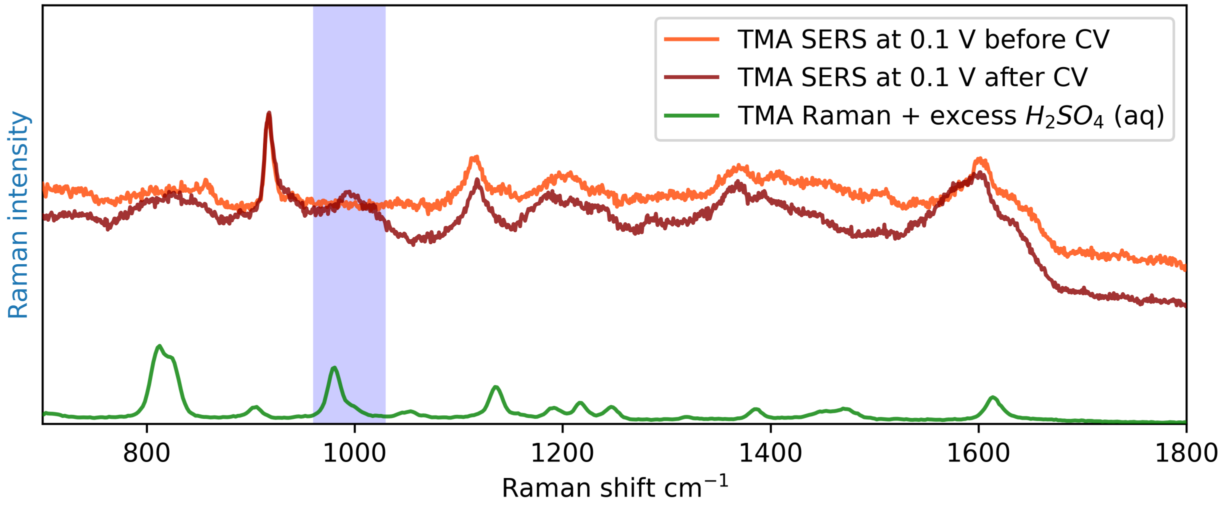

Figure S25: Raman spectra (λ_{exc} 785 nm) of **TMA** (1 mM) in CH_3CN, before (orange) and after (red) cyclic voltammetry between 0 V to 0.85 V. The spectrum of TMA in conc. H_2SO_4 (green) is shown for comparison showing the broad band at 1000 cm^{-1}. The band highlighted is assigned to the N-H stretch of **TMA−H$^+$**), observed also during cyclic voltammetry of **TMA** (Figure 9). Cyclic voltammetry was performed using a Ag/AgCl as reference, Pt as counter and roughened gold bead as working electrode
